# Purification and preparation of *Marchantia polymorpha* Auxin Response Factor 2 for phase separation studies

**DOI:** 10.1002/2211-5463.70313

**Published:** 2026-07-21

**Authors:** Bas Janssen, Robin Romein, Willy A. M. van den Berg, Simon Lindhoud, Jan Willem Borst, Carlo P. M. van Mierlo

**Affiliations:** ^1^ Laboratory of Biochemistry Wageningen University and Research The Netherlands

**Keywords:** auxin biology, Auxin Response Factor, biomolecular condensates, intrinsic disorder, protein purification

## Abstract

Eukaryotic cells compartmentalise to optimise their biochemistry. This occurs through membrane‐surrounded compartments, but also through membraneless organelles (MLOs). Many MLOs arise from Liquid–Liquid Phase Separation (LLPS) of proteins. To investigate proteins for their capacity to phase separate, as well as what properties this results in, purified protein is required for *in vitro* phase separation assays. We have observed that Auxin Response Factor 2 from the common liverwort *Marchantia polymorpha* (MpARF2) forms nuclear assemblies *in vivo*. Auxin Response Factors (ARFs) are transcription factors crucial for the regulation of gene expression in response to auxin, an essential signalling molecule in plants. It is currently unclear what causes the formation of MpARF2 assemblies and how they affect the protein's function. To decipher this, we present the procedures for purifying MpARF2 and preparing it for *in vitro* studies. The observed MpARF2 assemblies could arise by phase separation. The properties of phase‐separating proteins make their expression, purification and handling challenging; therefore, optimised procedures are required. Here, we show that the purification of soluble and monomeric full‐length MpARF2, as well as (truncated) variants, is possible using a three‐step purification procedure involving a protein solubility tag and buffers that suppress phase separation. We also describe the subsequent protein preparation that ensures reproducible phase separation of MpARF2 during *in vitro* experiments. These procedures pave the way for elucidating the phase separation properties of MpARF2 and the potential functional role of its assemblies while providing a protocol which can facilitate similar studies of other ARFs.

## Graphical abstract

We describe detailed protocols for the purification and preparation of *Marchantia polymorpha* Auxin Response Factor 2 (MpARF2). This protein is fused to an MBP solubility tag and an mNG fluorescent tag and is purified from *Escherichia coli*. The presented procedures make it possible to study MpARF2 assemblies, which could arise from phase separation.

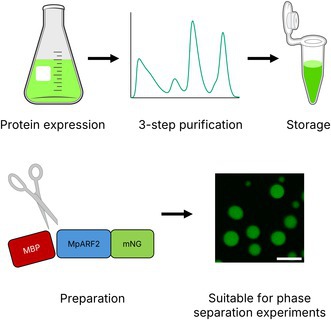

**Abbreviations**
(Mp)ARF: (*Marchantia polymorpha*) Auxin Response Factor
AuxRE: auxin response element
BCA: bicinchoninic acid
BSA: bovine serum albumin
CFE: cell‐free extract
*c*_sat_
: saturation concentration
CV: column volume
DBD: DNA‐binding domain
DTT: dithiothreitol
EDTA: ethylenediaminetetraacetic acid
EMSA: electrophoretic mobility shift assay
FPLC: fast protein liquid chromatography
HRV: human rhinovirus
IDP: intrinsically disordered protein
IDR: intrinsically disordered region
IPTG: Isopropyl β‐d‐1‐thiogalactopyranoside
LLPS: liquid–liquid phase separation
MBP: maltose‐binding protein
MLO: membraneless organelle
mNG: monomeric NeonGreen
MR: middle region
MWCO: molecular weight cut‐off
NAP: nuclear auxin pathway
Ni‐IMAC: Nickel‐Immobilised Metal Ion Affinity Chromatography
OPCA: octicosapeptide repeat, p40phox and budding yeast Cdc24p, atypical PKC‐interaction domain
PB1: Phox and Bem1
SD200‐I: Superdex 200 Increase
SD200PG: Superdex 200 Prep Grade
SDS/PAGE: sodium dodecyl sulphate‐polyacrylamide gel electrophoresis
SEC: size exclusion chromatography
SEC‐MALS: size exclusion chromatography with multi‐angle light scattering
TCEP: tris(2‐carboxyethyl)phosphine
TEV: tobacco etch virus
V_0_
: void volume

Eukaryotic cells compartmentalise to optimise and control their biochemistry. One way to achieve this compartmentalisation is by creating organelles surrounded by lipid membranes, such as the nucleus, endoplasmic reticulum, the Golgi apparatus, lysosomes or vacuoles. The membrane acts as a physical barrier to separate the in‐ and outside of these organelles. However, various organelles are not surrounded by a membrane, such as the nucleolus [1], Cajal bodies [2], stress granules [3] and P granules [4]. Such ‘membraneless organelles’ (MLOs) are also referred to as biomolecular condensates, referring to the mechanism by which they form: the spontaneous local concentration of biomolecules [5].

Biomolecular condensates are widely thought to form through Liquid–Liquid Phase Separation (LLPS) [4, 5, 6], a process whereby a solution with at least one solute will demix into two immiscible phases, a solute‐rich phase and a solute‐poor phase, both with liquid properties. In biomolecular condensates, the solute is a biomolecule, usually a protein, RNA or DNA, and the resulting solute‐rich phase is called a condensate. LLPS is highly concentration dependent, as condensates only form above a system‐specific saturation concentration (*c*_sat_) [7]. In addition, the saturation concentration strongly depends on environmental factors, such as temperature, pH or salinity [8].

Although biomolecular condensates form through phase separation, the underlying mechanisms vary considerably. Electrostatic interactions, hydrophobic effects, π‐π and cation‐π interactions can all contribute to condensate formation [5, 9], and are shaped by sticker‐spacer architecture and the presence of folded domains and disordered regions [10, 11]. As a result, the properties of condensates often differ. Condensates have different capacities to exchange molecules across their boundaries [12], to include or exclude certain molecules [13], to form or dissolve in response to stimuli [14, 15], to transition to a solid‐like state (also called ageing) [16] and have varying internal dynamics [17]. To understand the role of these properties in the function of condensates and MLOs, a combined approach of *in vitro* and *in vivo* studies is required.

To perform *in vitro* experiments with a phase‐separating protein, sufficient pure protein is required. A commonly used method to achieve the latter is to isolate a recombinant protein from a heterologous overexpression system. Phase‐separating proteins can be challenging to purify, as they are often aggregation‐prone, readily undergo interactions with other biomolecules and column materials, are highly susceptible to proteolysis, and can prematurely undergo phase separation, which can interfere with purification [18, 19, 20]. In addition, results sometimes differ between laboratories [18], and deviating from a specific preparation procedure can affect results [21]. Therefore, it is necessary to accurately document the purification and preparation procedures of the protein in question and to identify which elements are important for reliability and reproducibility of experiments. Here we report this for an Auxin Response Factor (ARF) protein from *Marchantia polymorpha*.

In plants, growth and development are regulated by the essential signalling molecule auxin. Response to auxin is primarily achieved by influencing gene expression through the Nuclear Auxin Pathway (NAP) [22]. Briefly, auxin promotes binding of Aux/IAA repressor proteins to TIR1/AFB, which is part of a Skp1, Cullin and F‐box (SCF) E3 ubiquitin ligase complex. This binding leads to the ubiquitination and subsequent degradation of Aux/IAA, thus releasing ARFs from repression and allowing them to modulate gene expression [23, 24]. ARFs are subdivided into three clades: A‐class ARFs, which activate gene expression, and B‐ and C‐class ARFs, which are generally regarded as repressors [25].

ARFs consist of three domains: an N‐terminal DNA‐binding domain (DBD), a Middle Region (MR) of variable length and a C‐terminal Phox and Bem1 (PB1) multimerisation domain. The DBD binds to DNA recognition sites called Auxin Response Elements (AuxREs) via its B3 domain and can dimerise via its bipartite dimerisation domain [26]. The PB1 domain can homo‐multimerise in a head‐to‐tail manner through interactions between the positively charged lysine and the negatively charged Octicosapeptide repeat, p40phox and budding yeast Cdc24p, atypical PKC‐interaction domain (OPCA) motif [27, 28]. The amino acid composition of the MR determines which class an ARF belongs to and thus whether an ARF will activate or repress gene expression [25, 29].

The NAP is studied in various model plants, many of which have complex NAPs with multiple copies of each NAP component. *Marchantia polymorpha*, a model bryophyte, is studied for its minimal NAP, as its genome encodes only one copy of each key component, including one copy of each ARF clade [30]. This is thought to represent the minimum complexity of an auxin response system that cannot be reduced further without loss of functional auxin response, except potentially its C‐class ARF, MpARF3 [30, 31]. The minimal NAP of *M. polymorpha* allows for research into the fundamental biochemical principles underlying the NAP and how these relate to the auxin response.

MpARF2, the B‐class ARF of *M. polymorpha*, forms visible assemblies at endogenous protein levels [32], but it is unclear why this happens and what properties these assemblies confer on the protein's function. To decipher this, we aim to apply a combination of *in vitro* and *in vivo* research.

The chitin‐affinity method previously used in our laboratory to purify ARF from *Arabidopsis thaliana* [33] did not work for MpARF2, because the protein is lost during the essential size exclusion chromatography (SEC) step (data not shown). Therefore, we developed other procedures to purify MpARF2 and prepare it for experiments. We fused the protein N‐terminally to a maltose‐binding protein (MBP) tag and C‐terminally to a monomeric NeonGreen(mNG)‐6xHis‐tag. Purification using Nickel Immobilised Metal Ion Affinity Chromatography (Ni‐IMAC), SEC and amylose affinity in 1 m NaCl, 10% glycerol, 20 mm Tris pH 8.0 allows to reliably obtain purified fusion protein (Fig. 1A). After removal of the MBP solubility tag and isolation of the monomer in an intermediate salt strength buffer (i.e. 500 mm NaCl, without glycerol), the protein is suitable for experiments to characterise its phase separation (Fig. 1B).

**Fig. 1:**
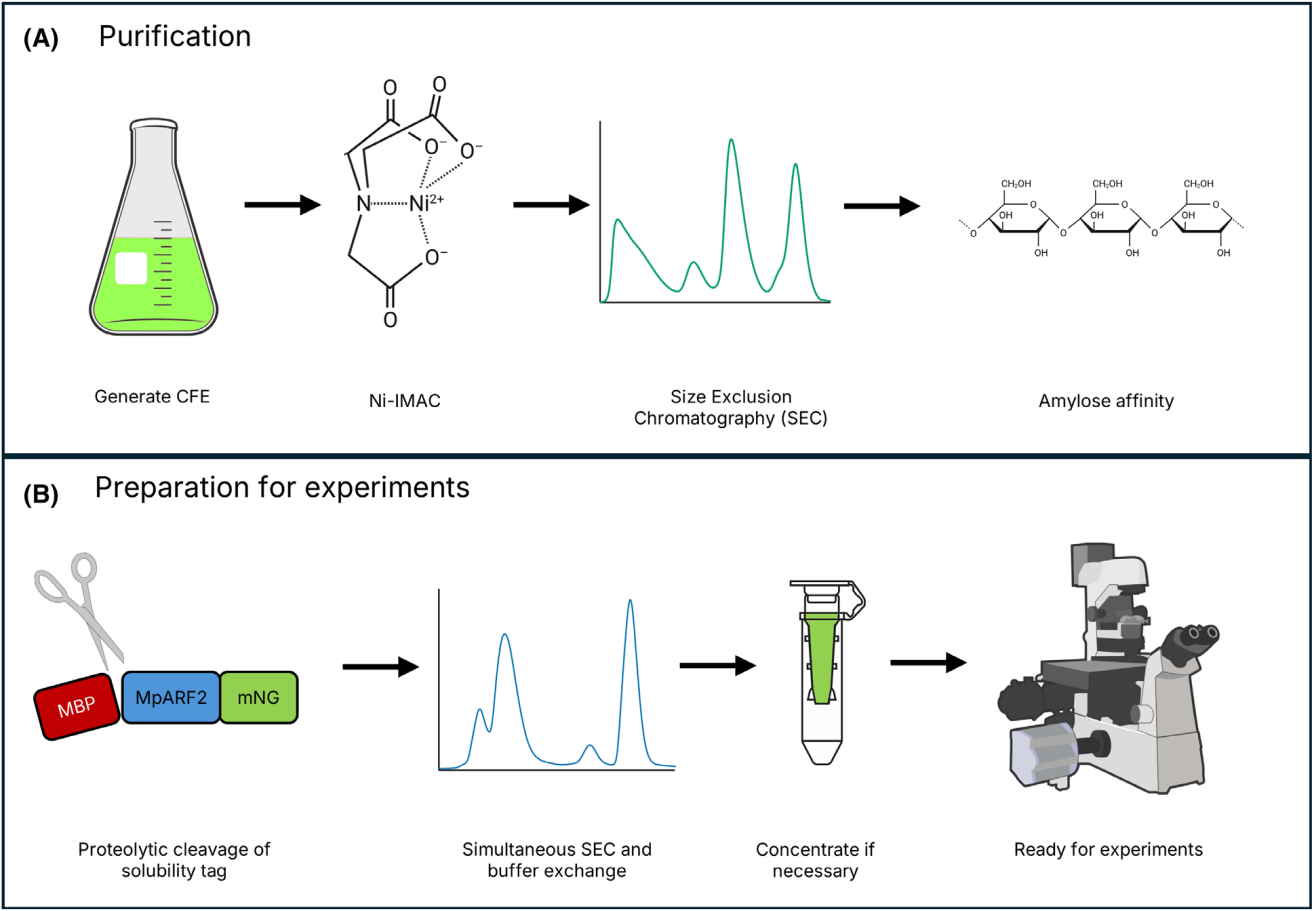
Schematic overview of purification (A) and sample preparation (B) workflows. (A) Recombinant protein is purified from cell‐free extract (CFE) by Ni‐IMAC, size exclusion chromatography (SEC) and amylose affinity steps. (B) Just before experiments, the protein is prepared by removing the solubility tag and then passing it through a small SEC column to isolate monomeric protein and exchange the buffer. The protein can then be used directly for experiments or, if necessary, concentrated first. Erlenmeyer, scissor and microscope illustrations from NIAID NIH BioArt Source (bioart.niaid.nih.gov/bioart).

This study provides detailed documentation on how to obtain and prepare MpARF2‐mNG and its (truncated) variants for phase separation experiments. We highlight essential steps during the purification and preparation of this protein as well as the pitfalls, sparing others the many challenges we encountered, and offer suggestions on how to maximise protein yield. Moreover, this documentation ensures that the experimental phase separation data of MpARF2‐mNG can be reliably reproduced by others. These procedures also provide a starting point for the purification and phase‐separation studies of other ARFs, which may ultimately lead to insights into the phase‐separation properties of ARFs and the potential functional role of ARF assemblies in plants.

## Materials

### Cloning

- Phusion™ High‐Fidelity PCR master mix (F531L; Thermo Fisher Scientific, Waltham, MA, USA)
- pET28a(+) plasmid (Novagen (Merck), Darmstadt, Germany)
- Primers ordered from IDT (Table S1)
- Template genes ordered from IDT (Table S2)
- Thermocycler
- GeneJET PCR Purification kit (K0701; Thermo Fisher Scientific)
- NEBuilder™ HiFi DNA assembly master mix (E2621L; NEB, Ipswich, MA, USA)
- Electrically competent DH5α *E. coli*
- Electroporation cuvettes
- Micropulser electroporator (no.: 1652100; Bio‐Rad, Hercules, CA, USA)
- Luria Broth (LB) medium and agar
- Kanamycin sulphate monohydrate (K0126; Duchefa, Haarlem, the Netherlands)
- Petri dish
- 15‐mL centrifuge tubes
- Shaker incubator
- Centrifuge
- Nucleospin Plasmid Mini kit (no.: 740588; Machery‐Nagel, Düren, Germany)
- EcoRI and HindIII restriction enzymes (ER0271/ER0501; Thermo Fisher Scientific)

### Protein production and generation of cell‐free extract (CFE)

- Luria broth (LB) agar
- Rosetta™ (DE3) *E. coli* (no.: 70954; Novagen (Merck))
- LB medium
- Kanamycin sulphate monohydrate (K0126; Duchefa)
- Chloramphenicol (C0113; Duchefa)
- Terrific broth (TB) medium (no.: 97063‐418; VWR, Radnor, PA, USA)
- Conical flask 250 mL
- Conical flasks 2 L
- Autoclave
- Petri dish
- Incubator
- Shaker incubator
- Isopropyl β‐d‐1‐thiogalactopyranoside (IPTG, 1 m solution in water) (Duchefa I1401)
- Ultracentrifuge
- 500‐mL centrifuge tubes
- 50‐mL centrifuge tubes
- Scale
- Lysis buffer A (in‐house production, see Table 1 for composition)
- Lysis buffer B (in‐house production, see Table 1 for composition)
- DNAse I, Grade II, from bovine pancreas (no.: 10104159001; Roche, Basel, Switzerland)
- cOmplete protease inhibitor cocktail tablets, EDTA‐free (no.: 04693132001; Roche)
- Sonicator (Q500; Qsonica, Newtown, CT, USA)
- Half‐inch (½″) diameter sonicator probe

**Table 1:** Purification buffers. All % concentrations are v/v. EDTA, ethylenediaminetetraacetic acid, TCEP, Tris(2‐carboxyethyl)phosphine.

Buffer	Composition
Lysis buffer A	40 mm Tris pH 8, 2 m NaCl, 20% glycerol, 4 mm MgCl_2_, 0.2% NP‐40
Lysis buffer B	20 mm Tris pH 8, 1 m NaCl, 10% glycerol, 2 mm MgCl_2_, 0.1% NP‐40
Ni‐Wash buffer	20 mm Tris pH 8, 1 m NaCl, 10% glycerol, 1 mm TCEP, 20 mm imidazole, 0.01% Tween‐20
Ni‐Elution buffer	20 mm Tris pH 8, 1 m NaCl, 10% glycerol, 1 mm TCEP, 500 mm imidazole, 0.01% Tween‐20
SEC buffer #1	20 mm Tris pH 8, 1 m NaCl, 10% glycerol, 1 mm EDTA, 1 mm DTT, 0.01% Tween‐20
Amylose elution buffer	20 mm Tris pH 8, 1 m NaCl, 10% glycerol, 1 mm EDTA, 1 mm DTT, 20 mm Maltose, 0.01% Tween‐20
SEC buffer #2	20 mm Tris pH 7.5, 500 mm NaCl, 1 mm EDTA, 1 mm DTT, 0.001% Tween‐20

### Protein purification

- Ni‐charged Nuvia™ Immobilised Metal Ion Affinity Chromatography (IMAC) resin (no.: 7800801; Bio‐Rad), or equivalent Ni‐IMAC resin
- 50‐mL screwcap tubes
- Microcentrifuge
- Serological pipette
- Pipetting balloon
- Gravity‐flow column compatible with peristaltic tubing
- Peristaltic pump and tubing
- Ni‐Wash buffer (in‐house production, see Table 1 for composition), filtered and degassed
- Ni‐Elution buffer (in‐house production, see Table 1 for composition), filtered and degassed
- Liquid nitrogen
- Dewar
- Ultra‐Low Temperature (ULT) freezer
- Äkta Pure 25, with multiple wavelength detector (MWD) (no.: 29018226; Cytiva, Marlborough, MA, USA)
- Fractionator (no.: 29011362; Cytiva)
- XK 26/100 column (no.: 28988951; Cytiva) packed with Superdex™ 200 Prep Grade resin (no.: 17104304; Cytiva) (HiLoad™ 26/1000 Superdex™ 200 PG) or equivalent prep‐grade size exclusion column
- SEC buffer #1 (in‐house production, see Table 1 for composition), filtered and degassed
- Whatman® Puradisc 30 syringe filters, 0.2 μm pore size (10462300; WHA)
- 50‐mL syringe with Luer lock
- Amicon® Ultra‐15 10 k molecular weight cut‐off (MWCO) (UFC901008; MilliporeSigma, Burlington, MA, USA)
- Superloop 10 mL (no.: 19758501; Cytiva)
- 15‐mL tubes compatible with fractionator
- Dithiothreitol (DTT, 1 m stock solution in water) (D1309; Duchefa)
- Äkta Start (no.: 29022094; Cytiva) (optional)
- 5 mL MBPTrap™ HP column (no.: 28918779; Cytiva) or equivalent amylose affinity column
- Amylose Elution buffer (in‐house production, see Table 1 for composition), filtered and degassed.
- Lo‐bind PCR strips
- Nanodrop
- Protein Lo‐bind 1.5‐mL microcentrifuge tubes
- Microcentrifuge
- Human Rhinovirus (HRV) 3C protease (SAE0045; Sigma‐Aldrich, St. Louis, MO, USA)
- Superdex™ 200 Increase 10/300 GL (no.: 28990944; Cytiva) or equivalent small‐scale size exclusion column
- SEC Buffer #2 (in‐house production, see Table 1 for composition), filtered and degassed
- 500‐μL injection loop
- Bovine serum albumin (BSA) (no.: 10735078001; Roche)
- Amicon® Ultra‐0.5 10 k MWCO (UFC501096; MilliporeSigma)

### Sodium dodecyl sulphate polyacrylamide gel electrophoresis (SDS/PAGE)

- 4× Bolt™ LDS Sample Buffer (B0007; Invitrogen, Waltham, MA, USA)
- 10× Bolt™ Sample Reducing Agent (B0009; Invitrogen)
- Mini Gel Tank (Invitrogen A25977)
- 20× Bolt™ MOPS SDS Running Buffer (B0001; Invitrogen)
- Bolt™ Bis‐Tris Plus Mini Protein Gels, 4–12% (NW04125BOXPR; Invitrogen)
- Power supply
- Bio‐Safe™ Coomassie Stain (no.: 1610786; Bio‐Rad)
- ChemiDoc XRS+

### Confocal microscopy

- Microscope slide
- Grace Bio‐Labs SecureSeal™ imaging spacer (GBL654008)
- Precision coverslip, thickness No. 1.5H (no.: 0107242; Marienfeld, Lauda‐Königshofen, Germany)
- Leica TCS SP8 × SMD or equivalent confocal laser scanning microscope

### Size exclusion chromatography with multi‐angle light scattering (SEC‐MALS)

- Ultrafree Centrifugal filter, 0.5 mL pore size 0.1 μm (UFC30VV; MilliporeSigma)
- 1260 Infinity II HPLC system (Agilent, Santa Clara, CA, USA)
- Superdex™ 200 Increase 10/300 GL (no.: 28990944; Cytiva)
- Merck MF‐Millipore Membrane Filter, 0.1 μm (no.: 10024450; Merck, Darmstadt, Germany)
- Running buffer: 20 mm Tris pH 8.0, 300 mm NaCl (in‐house production)
- Optilab™ 1090 differential refractive index detector (Wyatt technology, Santa Barbara, CA, USA)
- MiniDAWN™ 1065 MALS system (Wyatt technology)

### Electrophoretic mobility shift assay (EMSA)

- DNA probes PC019/PC020 (Table S2)
- Herring sperm DNA (D3159; Sigma‐Aldrich)
- Interaction buffer: 20 mm 4‐(2‐hydroxyethyl)‐1‐piperazineethanesulfonic acid (HEPES) pH 7.8, 50 mm KCl, 100 mm Tris pH 8.0, 2.5% glycerol, 1 mm DTT (in‐house production)
- Half‐strength Tris‐borate‐EDTA (TBE) buffer: 44.5 mm Tris, 44.5 mm boric acid, 1 mm EDTA (in‐house production)
- TopVision Agarose (R0492; Thermo Fisher Scientific)
- Gel electrophoresis tank and power supply
- Ettan DIGE imager (Cytiva)

## Method

### Cloning

All PCRs were performed using Phusion™ High Fidelity polymerase according to the manufacturer's instructions.

First, we generated a pET plasmid encoding the N‐terminal and C‐terminal tags, with a cloning site (MCS) in between. To this end, a pET28a(+) plasmid was linearised by PCR using primer pair BPJ030/BPJ031 (See Table S1 for primer sequences), MBP was amplified using primer pair BPJ002/BPJ029, and mNG was amplified using primer pair BPJ005/BPJ032 (See Table S2 for template sequences). These linear fragments, together with an MCS dsDNA oligo, were then cloned together using HiFi DNA Assembly (NEB) according to the manufacturer's instructions, yielding pET_MBP‐MCS‐mNG‐His.

For inserting coding sequences, the pET_MBP‐MCS‐mNG‐His vector was linearised by PCR using primer pair BPJ161/BPJ162. Full‐length MpARF2 (M1‐M878) and the K760S and D809A/D813A variants [34] were amplified by PCR using primer pair BPJ163/BPJ166. MpARF2‐DBD (M1‐F396) was amplified using primer pair BPJ163/165. For MpARF2^ΔMR^, DBD was amplified using primer pair BPJ061/BPJ163 and PB1 (K745‐M878) was amplified using primer pair BPJ062/BPJ166. The fragments were then inserted into the linearised vector using HiFi DNA Assembly (NEB) according to the manufacturer's instructions.

The coding sequences of MpARF2‐MR and MpARF1‐MR were inserted by linearising the pET_MBP‐MCS‐mNG‐His vector using EcoRI and HindIII restriction enzymes, amplifying MpARF2MR (S397‐N744) using primer pair BPJ013/BPJ014 and amplifying MpARF1‐MR (T370‐Q779) using primer pair BPJ121/BPJ122, and inserting the fragments into the linearised vector using HiFi DNA Assembly (NEB) according to the manufacturer's instructions.

All plasmids were sequenced by Nanopore sequencing (Plasmidsaurus).

### Protein production and generation of cell‐free extract (CFE)

Liquid media should be autoclaved in the flasks in which they will be used and then cooled to room temperature. Subsequently, all growth media are supplemented with 50 μg·mL^−1^ kanamycin and 10 μg·mL^−1^ chloramphenicol.

1. Transform pET_MBP‐MpARF2‐mNG‐His into Rosetta (DE3) *E. coli* and plate on Luria Broth (LB) agar with antibiotics. Grow overnight at 37 °C.
2. Pick a single colony using a sterile toothpick, pipette tip or inoculation loop, and inoculate into 50‐mL LB with antibiotics in a 250‐mL conical flask (See Tips and Tricks 1). Grow this culture at 37 °C while shaking at 180 rpm overnight.
3. Use this overnight culture, or preculture, to inoculate 3 L of Terrific Broth (TB) with antibiotics, divided across 6 × 2 L conical flasks, at a ratio of 1 mL of preculture to 100 mL of medium.
4. Place the conical flasks in an incubator and let the bacteria grow at 37 °C, 180 rpm until the optical density at 600 nm (OD_600_) reaches 0.6–0.8. This typically takes 2–3 h.
5. Add Isopropyl β‐d‐1‐thiogalactopyranoside (IPTG) to each flask to a concentration of 0.3 mm to induce expression of the protein of interest, lower the temperature and rotational speed of the incubator to 20 °C and 160 rpm, respectively, and incubate the cultures overnight (16–20 h).
6. After the overnight incubation, pellet the cells by centrifuging at 5000 ***g*** for 15 min in a centrifuge precooled to 4 °C (See Tips and Tricks 2).
7. Weigh the cells, and resuspend them in cold Lysis Buffer A, adding 1 mL of Lysis Buffer A for every gram of cell pellet (See Tips and Tricks 3). Keep the cells chilled during resuspension.
8. Measure the volume of the cell suspension and add another 1 mL of cold Lysis Buffer B for every 1 mL of suspension. Keep the suspension chilled.
9. Finally, for every 50 mL of cell suspension, add 10 mg DNAse I and 1 Roche cOmplete protease inhibitor cocktail tablet (or equivalent). Crush the tablet before adding it.
10. Lyse the cells using a sonicator for 2–3 min (See Tips and Tricks 4) at 80% amplitude, using a 5 s on/20 s off programme. Sonicate in an ice‐water bath to avoid overheating of the sample.
11. After sonication, clarify the lysate by centrifuging for 30 min – 1 h at 50 000 ***g*** at 4 °C (See Tips and Tricks 5).
12. Carefully decant the supernatant, hereafter designated cell‐free extract (CFE), into a conical flask or 50‐mL tubes. A 250‐mL flask is typically suitable. If the CFE is not used immediately, cover the flask and keep on ice until use.
13. Keep a small aliquot of the CFE (10 μL or more) for SDS/PAGE analysis.

### Purifying MBP‐MpARF2‐mNG from the CFE

All steps are carried out at room temperature (RT).

#### Step 1: Nickel IMAC

1. Gently resuspend Nuvia™ Ni‐IMAC resin (or equivalent, hereafter designated ‘Ni‐IMAC resin’) by swirling the bottle or using a glass stir rod.
2. Transfer 20 mL of slurry (containing 10 mL resin) to a 50‐mL tube and centrifuge at 7000 ***g*** for 10 min (note: 1000 ***g*** for 1 min should suffice).
3. Remove supernatant with a serological pipette and add Lysis Buffer B up to 50 mL. Resuspend the resin thoroughly and centrifuge again at 7000 ***g*** for 10 min (note: 1000 ***g*** for 1 min should suffice).
4. Remove supernatant again. Add Lysis Buffer B up to 20 mL and resuspend.
5. Add the resuspended Ni‐IMAC resin to the CFE. Incubate the CFE‐resin mixture with gentle agitation for 1 h at RT (see Tips and Tricks 6).
6. After incubation, transfer the CFE‐resin mixture to a gravity‐flow column that can hold at least 15 mL of volume. Connect the outlet to a peristaltic pump.
7. Using the peristaltic pump connected to the bottom of the column, draw the liquid through the column at 2–3 mL·min^−1^ until all resin has been collected. Keep adding CFE‐resin mixture as needed; do not let the resin run dry. Collect and keep the flow‐through for SDS/PAGE analysis.
8. Once all resin has been loaded, wash with 10 column volumes (CVs) Ni‐Wash Buffer, keeping the same flow rate as before.
9. Check whether the flow‐through is free of mNG by shining a light through the tubing. If absorbance or fluorescence analysis is an option, these are preferred. If the flow‐through is not yet free of mNG, keep washing in 1 CV intervals until it is.
10. Once the flow‐through is free of mNG, elute with 2.5 CVs of Ni‐Elution Buffer, or until the eluate is free of mNG.
11. Take a sample of the eluate (10 μL or more) for SDS/PAGE analysis.
12. Optional: To increase yield, the flow‐through may be re‐incubated with fresh resin, repeating the purification as described above (See Tips and Tricks 7).
13. The resin can be reused, but it must first be cleaned by stripping the nickel, then washed thoroughly with NaOH and finally the nickel must be reloaded. Refer to the resin manufacturer's instructions for such a protocol.

At this point, the purification can be paused overnight. If done so, the protein should be snap‐frozen in 100 μL aliquots and stored at −60 to −80 °C until the next step. To snap freeze a large sample, such as the Ni eluate, we slowly drip it directly into liquid nitrogen in a clean dewar, creating small frozen protein droplets which can be easily transferred to a 50‐mL tube to be stored at −60 to −80 °C. Note that a maximum of 25 mL of eluate will fit in a 50‐mL tube after freezing, due to air space between the frozen droplets. Also, be careful not to cap the 50 mL tube, as any liquid nitrogen inside will rapidly evaporate and may cause the tube to explode.

If purification is paused overnight, the end of Day 1 is a good time to start equilibrating the Size exclusion column (see Step 2).

#### Step 2: Size exclusion chromatography

1. Connect a HiLoad 26/1000 Superdex™ 200 PG (or equivalent, hereafter designated SD200PG) to an ÄKTA Pure 25 [or equivalent fast protein liquid chromatography (FPLC) system]. Equilibrate with SEC buffer #1, running as slowly as possible to minimise risk of resin compaction. We typically use a flowrate of 0.5–0.7 mL·min^−1^.
2. If purification has been interrupted overnight, thaw the Ni eluate and remove aggregates by filtering through a 0.22‐μm filter or by centrifugation at 21 000 ***g*** for 10 min at RT.
3. Concentrate the protein using a centrifugation filter with a 10 kDa Molecular Weight Cut‐off (MWCO) to the maximum loading volume of the SD200PG column or less (See Tips and Tricks 8, 9).
4. After the concentration step and after the SD200PG has been equilibrated, load the protein into a (super)loop and inject onto the column. We recommend using a superloop over a tubing‐based loop because the high viscosity of the protein solution in a tubing‐based loop leads to incomplete injection onto the column.
5. After injecting the protein, run the column for 1 CV with SEC buffer #1. Take fractions of 5–15 mL after passing the void volume (V_0_) of the column.
6. For quality control (QC) purposes, monitor A_280_, A_260_ and A_506_ simultaneously if possible. A_506_ is monitored because it is the A_max_ of the mNG tag, enabling the tracking of mNG‐containing proteins [35].
7. After passing the Ni eluate over the SD200PG column, pool the monomer fractions. Save all fractions for SDS/PAGE analysis, including small aliquots of the monomer fractions before pooling.
8. If the SD200PG column was equilibrated overnight with dithiothreitol (DTT) as the reducing agent, add 5 mm fresh DTT to the monomer fractions, mix well and incubate for 30 min before proceeding (See Tips and Tricks 10).

#### Step 3: Amylose affinity chromatography

1. Connect a 5‐mL MBPTrap™ HP column (or equivalent, hereafter designated ‘amylose column’) to a peristaltic pump or FPLC system.
2. Equilibrate the amylose column with fresh SEC buffer #1 (See Tips and Tricks 11).
3. Load the pooled monomer fraction onto the amylose column at a flowrate of 1–2 mL·min^−1^. Collect and keep the flow‐through for SDS/PAGE analysis.
4. Wash with three CVs of fresh SEC buffer #1, or until no protein is detectable when the column is washed.
5. Elute with two CVs of fresh Amylose Elution Buffer. It is advisable to collect the eluate in fractions of 1 mL (0.2 CV).
6. Measure the protein concentration of the eluate by measuring the A_506_ (See Tips and Tricks 12) using the ε_506_ of mNG, which is 116 000 m ^−1^ cm^−1^ [35]. Molarities can be converted into mg·mL^−1^ using molecular weights listed in Table 2.
7. Pool the samples with the highest protein concentrations (typically 3 mL in total), snap freeze in 100 μL aliquots and store at −60 to −80 °C.

**Table 2:** Protein yields after purification. Predicted molecular weights, predicted pIs and yields after the SD200PG step and after complete purification of maltose‐binding protein (MBP)‐tagged protein are given per protein variant. Molecular weights (Mws) and iso‐electric points (pIs) are calculated by using the Iso‐Electric point Calculator (IPC) [46]. For the pI values we used the IPC_protein pI. Calculated Mw values are rounded to the nearest integer. Yield ranges are shown as mean ± standard deviation (MpARF2: *n* = 5, MpARF2‐MR: *n* = 3, MpARF2^K760S^, MpARF2^D809A/D813A^, MpARF2DBD: *n* = 2, MpARF2^ΔMR^, MpARF1‐MR: *n* = 1).

Protein	Mw (Da)	pI	Yield after complete purification of MBP‐mNG‐tagged protein	Yield after SD200PG step
**MpARF2** (untagged)	96 421	6.87	1.13 ± 0.22 mg·L^−1^ culture	7.82 ± 0.82 mg·L^−1^ culture
MBP‐ and mNG‐tagged	168 544	6.03		
mNG‐tagged	126 485	6.72		
**MpARF2** ^**K760S**^ (untagged)	96 380	6.74	0.89 ± 0.21 mg·L^−1^ culture	18.1 ± 2.5 mg·L^−1^ culture
MBP‐ and mNG‐tagged	168 503	5.99		
mNG‐tagged	126 444	6.63		
**MpARF2** ^**D809A/D813A**^ (untagged)	96 333	7.13	0.14 ± 0.03 mg·L^−1^ culture	2.12 ± 0.17 mg·L^−1^ culture
MBP‐ and mNG‐tagged	168 456	6.11		
mNG‐tagged	126 397	6.92		
**MpARF2‐MR** (untagged)	37 969	8.39	2.76 ± 0.20 mg·L^−1^ culture	16.8 ± 2.4 mg·L^−1^ culture
MBP‐ and mNG‐tagged	110 091	6.05		
mNG‐tagged	68 033	7.73		
**MpARF2‐DBD** (untagged)	43 921	6.96	0.50 ± 0.11 mg·L^−1^ culture	30.6 ± 9.84 mg·L^−1^ culture
MBP‐ and mNG‐tagged	116 191	5.85		
mNG‐tagged	74 132	6.70		
**MpARF2** ^**ΔMR**^ (untagged)	58 618	6.00	0.10 mg·L^−1^ culture	5.20 mg·L^−1^ culture
MBP‐ and mNG‐tagged	130 887	5.68		
mNG‐tagged	88 828	6.15		
**MpARF1‐MR** (untagged)	44 635	5.81	1.42 mg·L^−1^ culture	5.67 mg·L^−1^ culture
MBP‐ and mNG‐tagged	116 905	5.55		
mNG‐tagged	74 846	6.14		

Protein purity should be checked by SDS/PAGE analysis (see below).

Using this protocol, one can reliably obtain approximately 1 mg of MBP‐MpARF2‐mNG per litre of cell culture medium.

### Protein preparation prior to experiments

1. Thaw up to 500 μL of purified MBP‐MpARF2‐mNG. Centrifuge at 21 000 ***g*** for 10 min at RT to remove any aggregates.
2. Measure the protein concentration and add 1 μg human rhinovirus (HRV) 3C protease (hereafter ‘3C protease’) per 1 nmol protein.
3. Do not concentrate the protein prior to adding the 3C protease. If the protein concentration is too high, phase separation will occur prematurely, even in a solution of 1 m NaCl and 10% glycerol. Under these conditions, we observed that MpARF2‐mNG undergoes premature phase separation at a concentration of 8 μm or higher. Generally, we keep the protein concentration at 6 μm or lower when adding 3C protease (See Proof‐of‐principle section).
4. After addition of 3C protease, incubate at RT for 2 h (See Tips and Tricks 13). Under these conditions, do not incubate for more than 2 h.
5. Meanwhile, equilibrate a Superdex™ 200 Increase 10/300 GL column (or equivalent analytical SEC column, hereafter designated SD200‐I) with SEC buffer #2. Connect the column to an ÄKTA Pure 25 or equivalent FPLC system and equilibrate by running with 1.5–2 CVs at a flowrate of 0.5 mL·min^−1^.
6. While the SD200‐I column is equilibrating, clean a 500‐μL injection loop by loading with 0.5 m NaOH. Incubate for a minute, then rinse well with MilliQ.
7. Optional: Load the injection loop with 500 μL of a 1% (w/v) bovine serum albumin (BSA) solution in SEC buffer #2. Incubate for 1 h or until the column is equilibrated (See Tips and Tricks 14). Do not inject into the column.
8. Wash the injection loop with SEC buffer #2. If BSA was loaded before, wash with at least 10 mL and monitor for release of BSA (e.g. by setting the FPLC system to inject the contents of the loop and bypass the column straight to a UV–Vis detector measuring A_280_). Wash until no more BSA is released.
9. After the 3C and MBP‐MpARF2‐mNG mixture has incubated for 2 h, immediately load it into the loop and inject it onto the SD200‐I column. Run for 1 CV with SEC buffer #2 (Table 1). Start taking 0.25–0.5 mL fractions after the V_0_ of the column (~ 7 mL) has been passed.
10. For QC purposes, monitor A_280_, A_260_ and A_506_.
11. MpARF2‐mNG monomer elutes at around 10 mL. To ensure reproducibility of experiments, only collect the fractions corresponding to the latter half of the monomer peak. The first half is less pure and sometimes contains larger particles, probably originating from the tail of species present at an elution volume of ~ 8.75 mL.
12. Save aliquots of all fractions for SDS/PAGE analysis
13. If the protein concentration of the collected fractions is sufficient, they can directly be used for experiments.
14. If the MpARF2‐mNG concentration is not sufficient, concentrate at RT using Amicon 10 kMWCO 0.5 mL concentration filters.
15. After concentrating to a total volume of ~ 40 μL or less, transfer to a Protein Lo‐Bind Eppendorf tube and determine the protein concentration by measuring A_506_. A loss of up to 75% of protein during concentration is consistently observed.

After preparing the protein as described above, it is ready for *in vitro* (phase separation) experiments. MpARF2‐mNG should be used immediately or stored at RT for a maximum of 1 h prior to experiments. The protein should never be cooled down, (snap‐)frozen or otherwise stored for extended periods prior to experimentation, as this will compromise the homogeneity of the sample. Protein purity should be routinely checked by SDS/PAGE analysis (see below) and nucleic‐acid contamination should be checked by measuring the A_260_/A_280_ ratio.

### SDS/PAGE

For SDS/PAGE purposes, the protein concentrations of impure samples were estimated by measuring A_280_, assuming 1 abs ~ 1 mg·mL^−1^. In the case of purified protein, protein concentrations were determined by measuring A_506_ as described above. Samples were mixed well with Bolt™ 4× LDS Sample Buffer and Bolt™ 10× Sample Reducing agent and subsequently incubated at 95 °C for 1 min. Samples were then loaded on Bolt™ Bis‐Tris 4–12% Plus Mini Protein Gels. CFE and Ni flow‐through samples were diluted 100×, after which 25 μL was loaded. For partially purified samples and Superdex 200 PG fractions, 5 μg was loaded. For purified protein, 0.5–1 μg was loaded. Gel electrophoresis was then performed according to the manufacturer's instructions, after which the gels were stained with Bio‐Safe™ Coomassie Stain according to the manufacturer's instructions. The gels were then imaged with a Chemidoc XRS+ against a white background.

### Confocal microscopy

To visualise whether samples contained microscopic protein structures, 5 μL of sample to be investigated was transferred to a microscopy slide with a Secureseal 8‐well imaging spacer. After filling the wells, a coverslip was added, and the whole slide was inverted and left for 15 min to let potential structures settle to the bottom.

Solutions were imaged using a confocal laser scanning microscope [Leica TCS SP8 x Single Molecule Detection (SMD)]. Samples were placed over a 63× water immersion objective. mNG was excited with a pulsed White Light Laser (WLL) at 488 nm and emission was detected with hybrid detectors (HyD) between 515 and 545 nm. Images were taken at the bottom of the well, at the coverslip.

### SEC‐MALS (optional)

To determine the oligomeric state of proteins, SEC‐MALS was performed as described previously [36]. SEC‐purified MBP‐MpARF2‐mNG fractions were filtered using a Ultrafree‐MC 0.1 μm centrifugal filter. Fifty microlitres of the filtered fractions was then run on a Superdex 200 Increase 10/300 GL column connected to a 1260 Infinity II HPLC system. The column was equilibrated with 20 mm Tris pH 8, 300 mm NaCl buffer, which had been filtered with a 0.1 μm Membrane Filter. Molecular mass of eluted sample was determined by Multi‐Angle Light Scattering with an Optilab 1090 Differential Refractive Index detector and a miniDawn 1065 MALS system.

### EMSA (optional)

To test the DNA binding of purified MpARF2 protein, samples were prepared and EMSAs were performed as described in ref [37]. 5′‐Cy5‐labelled sense single‐stranded IR7 DNA probe and its unlabelled antisense complement (PC019/020, Table S1) were annealed by heating at 95 °C for 5 min followed by a slow cooling down in a thermocycler overnight. MpARF2‐DBD used for this experiment was purified using chitin affinity as described previously [30, 38].

## Tips and tricks

1. TB can also be used for the preculture. However, we have noticed that a preculture grown in TB can sometimes prematurely induce expression of the protein of interest, particularly if the culture is poorly aerated. Therefore, we prefer to grow the preculture in well‐aerated LB to minimise the risk of premature induction. Additionally, 1% glucose may be added to the preculture to minimise leaky expression [39].
2. The cell pellets can be frozen at −80 °C for later use.
3. Mix Lysis Buffer A well before use.
4. Sonicate 2 min for 20–25 g cells, 3 min for 25–40 g cells.
5. Centrifuging for 30 min is sufficient to clarify the lysate, but the pellet may detach from the tube when decanting the supernatant. We typically centrifuge for 1 h.
6. The suspension can be gently agitated by rotating the head over the bottom, on a roller, or by using a motor‐driven glass stirring rod. Never use a magnetic stirrer; the abrasion will damage the resin.
7. The drawback of re‐incubating is that most of the additional yield consists of aggregates/multimeric complexes, which can complicate purification.
8. If the eluate concentration is already high, it may be useful to use a 50 kDa or 100 kDa MWCO filter to save time concentrating.
9. The maximum sample volume of the HiLoad 26/1000 Superdex 200 PG is 13 mL. However, to ensure good separation, we typically concentrate the protein to 5 mL.
10. When DTT is not added to the SEC samples, the binding of MBP‐MpARF2‐mNG to the MBPTrap™ HP column is especially poor. We suspect that the weak binding is due to the formation of intramolecular disulfide bonds that hinder the accessibility to MBP. It may be worthwhile to replace the DTT reducing agent in SEC Buffer #1 with TCEP to prevent this.
11. For the same reason as above, the DTT in SEC buffer #1 for use on the MBPTrap™ HP column should be fresh. The buffer can be prepared and filtered in advance without DTT. DTT can then be added from a filtered stock solution just before use.
12. Other methods of measuring protein concentration, such as Bradford or bicinchoninic acid (BCA) assay, can also be used. However, we find that measuring the A_506_ provides an accurate and quick measure of purified protein concentration (Fig. S1).
13. If shorter 3C protease incubation times are desired, higher ratios of 3C protease to protein can be used. Test optimal cleavage prior to use for experiments.
14. Coating the injection loop with BSA prior to loading slightly increases yield.

## Proof‐of‐principle

### MpARF2—purification

**Fig. 2:**
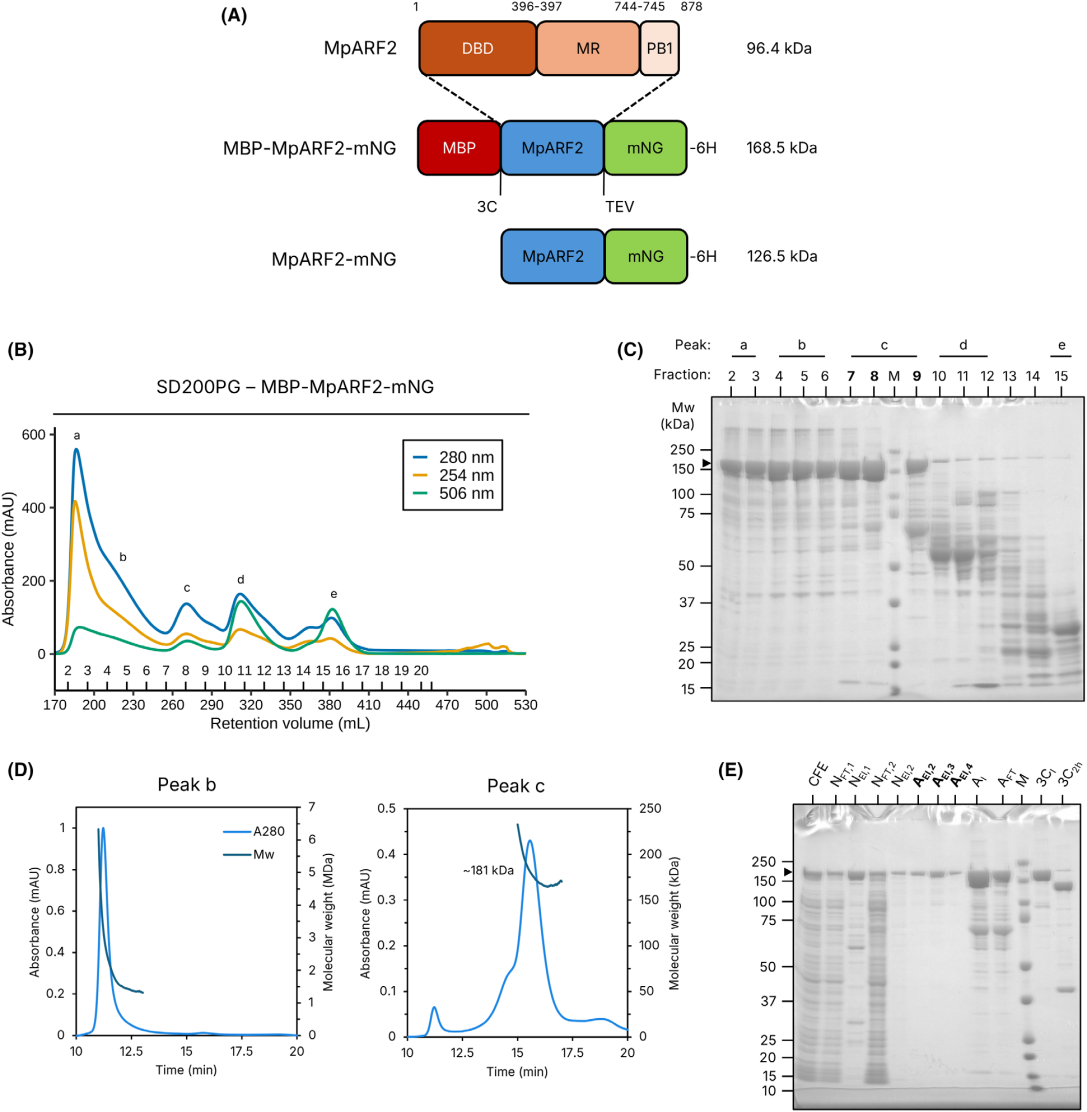
Purification of MBP‐MpARF2‐mNG. (A) Schematic representation of MpARF2 domains with their amino acid boundaries, and of maltose‐binding protein (MBP)‐tagged and MBP‐free MpARF2‐mNG, with corresponding molecular weights. 3C: HRV 3C protease site; TEV: TEV protease site; 6H: 6xHistidine tag. (B) UV–Vis chromatogram of SD200PG size exclusion chromatography (SEC) run of MBP‐MpARF2‐mNG Ni eluate. V_0_ = ~ 170 mL, CV = ~ 530 mL. MBP‐MpARF2‐mNG monomer elutes around 270 mL. Inner tick marks on the x‐axis indicate the starting points of fractions. Fraction 20 ends at the last tick mark. (C) SDS/PAGE gel of the SD200PG fractions. Fractions selected for subsequent amylose affinity purification are in bold. M: Molecular weight marker. (D) Size exclusion chromatography with multi‐angle light scattering (SEC‐MALS) analysis of monomer peak c and multimer peak b. Note that the molecular weight scales differ by an order of magnitude between the left and right panel. (E) SDS/PAGE gel of all non‐SEC samples. Purified protein samples after amylose affinity purification are highlighted in bold. 3C_2h_, sample after 2 h 3C incubation; 3C_I_, sample prior to adding 3C protease; A_El_, amylose eluate; A_FT_, amylose flow‐through; A_I_, amylose input; CFE, cell‐free extract; M, molecular weight marker; N_El_, nickel eluate; N_FT_, nickel flow‐through. Black arrowheads indicate the position of the protein of interest in (C and E). B, C and E are representative data from dozens of purifications. Panels in D are representative chromatograms from *n* = 2 measurements, the given molecular weight is an average from these measurements.

We fused MpARF2 N‐terminally to an MBP tag and C‐terminally to an mNG‐6xHis‐tag. The mNG fluorescent tag enables the visualisation of the protein during purification and confocal microscopy. We also inserted a 3C protease cleavage site between the MBP tag and MpARF2, as well as a tobacco etch virus (TEV) protease cleavage site between MpARF2 and the mNG tag, for separate proteolytic removal of each tag (Fig. 2A). After production in Rosetta™ (DE3) *E. coli*, we purify the fusion protein in 1 m NaCl, 10% glycerol and 20 mm Tris pH 8.0. Attempts to purify MBP‐MpARF2‐mNG in this buffer containing 0.5 m NaCl and no glycerol were unsuccessful.

After lysis of *E. coli* and the first purification step (i.e. Ni‐IMAC), four to five chromatography peaks are observed when passing the Ni eluate over the Superdex 200 PG size exclusion column (hereafter ‘SD200PG’) (Fig. 2B). We label these peaks a‐e.

Fractions corresponding to peaks a‐c all primarily contain MBP‐MpARF2‐mNG, as SDS/PAGE analysis mostly shows the presence of a band at ~ 170 kDa (Fig. 2A,C). We therefore reason that peak c represents monomeric MBP‐MpARF2‐mNG, as it is the last‐eluting peak that still primarily contains MBP‐MpARF2‐mNG. To verify this, we analysed fractions corresponding to peak c by SEC‐MALS, which reveals that these fractions indeed contain protein with a molecular weight of ~ 181 kDa (Fig. 2D, peak c), roughly corresponding to the theoretical molecular weight of MBP‐MpARF2‐mNG of 169 kDa (Table 2). The fractions corresponding to peak c (i.e. Fractions 7–9, Fig. 2B,C) are pooled for further purification.

Fractions corresponding to peaks a, d and e are discarded, as peak a elutes in the void volume (V_0_) and thus contains large aggregates, and contains a nucleic‐acid contaminant (i.e. A_280_/A_254_ ratio < 2). Peaks d and e contain C‐terminal proteolytic degradation products of MBP‐MpARF2‐mNG (Fig. 2C), as evidenced by their A_506_ (Fig. 2B) indicating the presence of the mNG tag, and by the low intensity of the ~ 170 kDa band corresponding to full‐length MBP‐MpARF2‐mNG (Fig. 2C). We note that the peak areas at 506 nm of peaks d and e are much larger than that of peak c, indicating that most of the MBP‐MpARF2‐mNG is cleaved by proteases. As we use protease inhibitors during MBP‐MpARF2‐mNG purification, we judge that this cleavage likely already occurs within *E. coli*.

To check whether peak b might represent a dimeric or a similarly small oligomeric species of MBP‐MpARF2‐mNG, we used SEC‐MALS, which reveals large multimeric species on the order of MDa (Fig. 2D, peak b). Fractions corresponding to peak b are therefore discarded. Interestingly, MDa‐sized species should elute in the V_0_ of the SD200PG column, since their size exceeds the separation range of this column (M_r_ ~ 600 kDa). We therefore hypothesise that MBP‐MpARF2‐mNG interacts weakly with the SD200PG resin, increasing its elution volume. This hypothesis is further supported by the presence of a band of ~ 170 kDa, corresponding to MBP‐MpARF2‐mNG, in all SEC fractions, even in later fractions that should correspond to a protein with a much smaller M_r_ (i.e. Fractions 11–15, Fig. 2C).

To further purify and simultaneously concentrate MBP‐MpARF2‐mNG, we passed the protein corresponding to peak c (Fig. 2B,C Fractions 7–9) over an amylose column. The collected flow‐through still contains MBP‐MpARF2‐mNG, as a 168.5 kDa band is clearly visible by SDS/PAGE (Fig. 2E, compare A_I_ and A_FT_), indicating that the protein binds relatively poorly to the amylose column. Re‐loading the flow‐through onto the column results in negligible to no binding (data not shown). Other than ensuring that the disulfide bonds are reduced properly, we could not improve MBP‐MpARF2‐mNG binding by, for example, lowering the concentration of glycerol used (data not shown).

After amylose affinity purification of the monomer‐containing protein fractions, we obtain 1.13 ± 0.22 mg of MBP‐MpARF2‐mNG per litre of cell culture medium (Table 2). Purity of this sample is confirmed by SDS/PAGE analysis (Fig. 2E, A_El_ lanes). Purifications of MBP‐MpARF2‐mNG are reproducible, with similar yields of comparable purity.

We used an EMSA to verify the DNA binding of the purified MBP‐MpARF2‐mNG. A correctly folded DBD should bind to repeat AuxREs [26], and a correctly folded PB1 domain should increase the affinity of MpARF2 for DNA [33]. Because MBP‐MpARF2‐mNG binds to an inverted double repeat of the TGTCGG AuxRE (IR7) with higher affinity than the isolated DBD domain of MpARF2 (MpARF2‐DBD) (Fig. S3), we infer that the DBD and PB1 exhibit their expected functions and are therefore correctly folded.

### MpARF2—Preparation for experiments

While we succeeded in purifying MBP‐MpARF2‐mNG, this protein is not yet suitable for phase separation experiments. The MBP solubility tag will interfere with phase separation and thus needs to be proteolytically removed by HRV 3C protease. In addition, we perform the phase separation assays at physiological‐like conditions (i.e. 125 mm NaCl, pH 7.5). We reasoned that straight dilution from the purification buffer (i.e. 1 m NaCl, 10% glycerol, pH 8.0) would be a too abrupt change and therefore opt to first bring the protein solution to an intermediate salt concentration (i.e. 500 mm NaCl). Finally, we wanted to ensure that there are no multimeric species present in the protein stock. To address these issues, we begin with a proteolytic cleavage step followed by SEC to obtain monomeric, MBP‐free protein in an intermediate strength salt buffer (SEC buffer #2, Table 1). The SEC step has the added benefit of removing free MBP and leftover protease after cleavage of the MBP tag.

While testing the proteolytic cleavage of the MBP tag, we noticed that MpARF2 also contains a secondary cleavage site for 3C protease (Fig. S2A). The protease targets this site with lower affinity than the primary cleavage site located between MBP and MpARF2. Therefore, we assayed MBP‐MpARF2‐mNG to determine an optimal incubation time and ratio of 3C protease to protein, maximising cleavage of the primary site and minimising cleavage of the secondary site. We chose a ratio of 1 μg 3C protease per nmol MBP‐MpARF2‐mNG and incubated for 2 h at room temperature (Fig. 2E, Fig. S2A). Adding more 3C protease or incubating longer increases secondary cleavage.

**Fig. 3:**
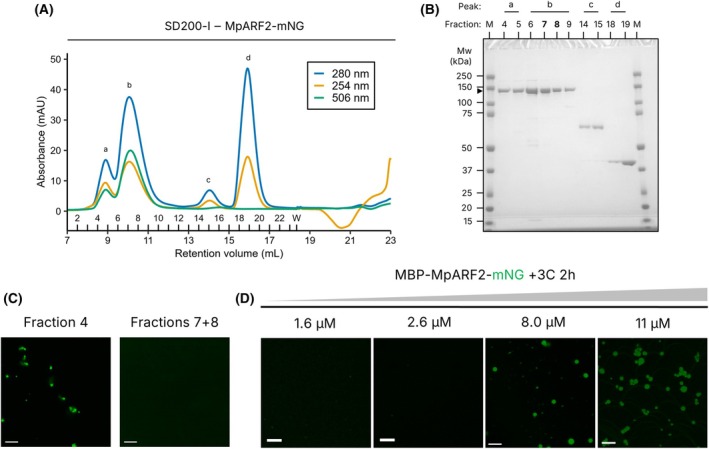
MpARF2‐mNG can phase‐separate prematurely during preparation for experiments. (A) UV–Vis chromatogram of SD200‐I size exclusion chromatography (SEC) run of 3C‐treated MBP‐MpARF2‐mNG. V_0_ = ~ 7 mL, CV = ~ 23 mL. Inner tick marks on the x‐axis indicate the starting points of fractions. Only even fractions are labelled. W = waste. (B) SDS/PAGE gel corresponding to SD200‐I run. Numbers correspond to fraction numbers. Fractions chosen for experiments are highlighted in bold. M, molecular weight marker. (C) Confocal microscopy images of Fraction 4 (approximately 100 nm MpARF2‐mNG) and of pooled Fractions 7 and 8 concentrated to 4 μm MpARF2‐mNG. (D) Confocal microscopy images of 3C‐treated samples of MBP‐MpARF2‐mNG at various concentrations, in 20 mm Tris pH 8, 1 m NaCl, 10% glycerol, 1 mm EDTA, 1 mm DTT. Scale bars (C, D): 5 μm. A, B and the right panel of C are representative data from dozens of purifications, D and the left panel of C are representative of *n* = 2 measurements.

After incubation of MBP‐MpARF2‐mNG with 3C protease, we use the analytical Superdex 200 Increase (hereafter ‘SD200‐I’) column to obtain monomeric MpARF2‐mNG in the intermediate salt strength buffer and to remove contaminants. The SD200‐I run of the MpARF2‐mNG cleavage mixture yields four peaks (Fig. 3A, peaks a–d). Fractions corresponding to peak a are discarded, as despite being pure as tested by SDS/PAGE (Fig. 3B), they have a low A_280_/A_254_ ratio (i.e. < 2), indicating contamination by nucleic acids. In addition, Fraction 4, which corresponds to the maximum of peak a, is an inhomogeneous solution with micron‐sized structures of MpARF2‐mNG as determined by confocal microscopy (Fig. 3C). Fractions corresponding to peaks c and d are also discarded, because they contain contaminants (secondary 3C cleavage product and free MBP, respectively (Fig. 3B)).

Peak b corresponds to monomeric MpARF2‐mNG. However, this peak partially overlaps with peak a (Fig. 3A). Knowing that peak a contains microscopic structures of MpARF2‐mNG (see Fig. 3C), we discard Fraction 6, corresponding to the first half of peak b, and use only Fractions 7 and 8, corresponding to the latter half of peak b. Fractions 7 and 8 are pure by SDS/PAGE analysis (Fig. 3B). After pooling and concentrating, confocal microscopy shows that the protein solution is homogeneous (Fig. 3C). We therefore conclude that Fractions 7 and 8 are suitable for phase separation experiments with MpARF2‐mNG. To ensure reproducibility during experiments, the MpARF2‐mNG stock solutions are regularly checked for absence of microscopic structures by confocal microscopy, in addition to routine the purity analysis by SDS/PAGE and spectrophotometry.

During the concentration of Fractions 7 and 8 we noticed that the protein yield after concentrating is lower than expected. Comparing the starting fractions and the concentrated protein stock shows that approximately 75% of the MpARF2‐mNG is lost (Table 3). The flow‐through of the concentration columns is not fluorescent (data not shown), indicating that the protein is not lost through leaky filters, but rather remains adhered to the concentration filters. We have not been able to resolve this issue.

**Table 3:** Recovery of MpARF2‐mNG variants/truncations after concentration. The stock volumes are estimates based on how much we pipetted from the filters. The MpARF2^ΔMR^ starting material was divided over two filters, which likely increases the margin of error in our volume estimation, leading to a relatively large error in recovery (i.e. 108%).

MpARF2‐mNG variant	Yield preconcentration (moles)	Stock concentration (μm)	Stock volume (μL)	Yield postconcentration (moles)	Recovery (%)
WT	5.996E‐10	3.60	40	1.44E‐10	24.0
K760S	3.209E‐10	6.91	40	2.76E‐10	86.1
D809A/D813A	1.390E‐10	3.29	40	1.32E‐10	94.7
MpARF2‐DBD	1.363E‐09	41.47	30	1.24E‐09	91.3
MpARF2^ΔMR^	6.720E‐10	10.6	30	3.18E‐10	108
		13.7	30	4.11E‐10	
MpARF2‐MR	6.216E‐10	18.4	30	5.52E‐10	88.8

Yield of monomeric MpARF2‐mNG is also limited by the maximum sample volume of the SD200‐I (i.e. 500 μL). We therefore attempted to concentrate the MBP‐MpARF2‐mNG/3C mixture before loading it onto the SD200‐I. However, this resulted in significant loss of protein because MpARF2‐mNG sticks to the concentration column. Instead, we concentrated MBP‐MpARF2‐mNG before adding 3C protease. However, when MBP‐MpARF2‐mNG is concentrated to 8 μm or higher, 3C cleavage leads to turbid samples (data not shown), which according to confocal microscopy is due to phase separation (Fig. 3D). The *c*_sat_ of MpARF2‐mNG under these conditions is therefore below 8 μm. Notably, this premature phase separation occurs in 1 m NaCl, 10% glycerol, pH 8.0, since the 3C incubation is carried out in amylose elution buffer (Table 1). To prevent premature phase separation of MpARF2‐mNG during protein preparation, we therefore keep its concentration well below this potential *c*_sat_ value. This means that we never concentrate the protein above 6 μm prior to 3C cleavage. If the concentration of MBP‐MpARF2‐mNG is higher than 6 μm, we dilute it to 6 μm before adding 3C protease.

Despite the limitations on protein yield described above, the handling procedure provides sufficient high‐quality protein for phase separation experiments. After performing the preparation procedure with up to 500 μL of a 6‐μm solution of MBP‐MpARF2‐mNG, we typically obtain approximately 30 μL of a 4‐μm solution of MpARF2‐mNG, which is sufficient for most experiments.

To ensure parity between experiments using MpARF2‐mNG and MpARF2‐mNG variants/truncations, the same purification and preparation protocols as discussed above are applied to all these variants/truncations. Below, we outline some notable deviations and observations per protein variant/truncation.

### PB1 variants of MpARF2—Purification and preparation for experiments

The PB1 domain homo‐multimerises in a head‐to‐tail fashion, through interactions between the invariant lysine on one interface and the OPCA motif on the other [27, 28]. MpARF2^K760S^ and MpARF2^D809A/D813A^ are variants of MpARF2 with altered interaction interfaces of the C‐terminal PB1 multimerisation domain. The K760S mutation targets the invariant lysine, and the D809A/D813A mutation targets the OPCA motif. As a result of these mutations, PB1 multimerisation is prevented [27].

**Fig. 4:**
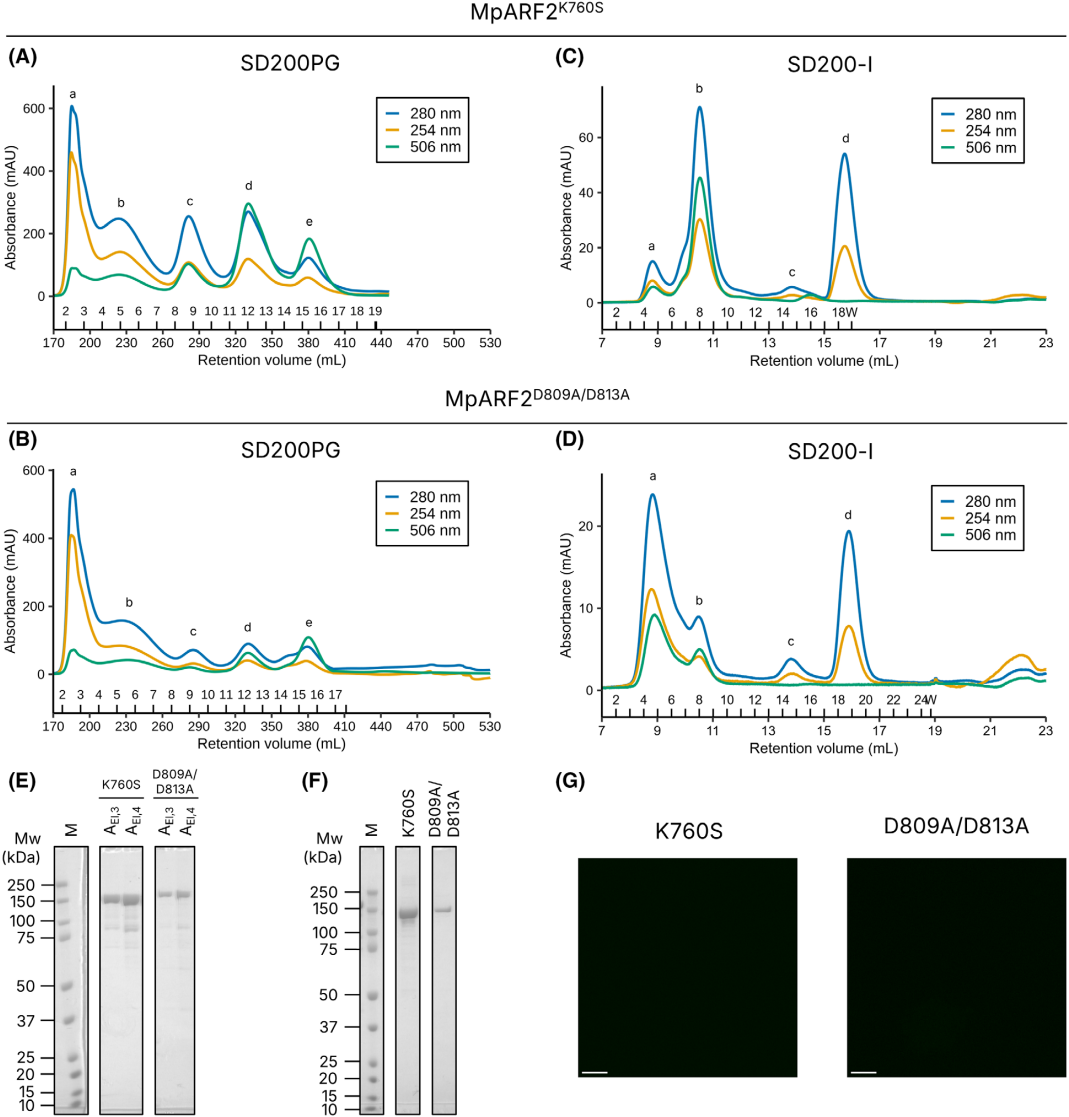
Purification and preparation of MpARF2 PB1 variants. (A, B) UV–Vis chromatograms of SD200PG size exclusion chromatography (SEC) runs of the K760S variant (A) and the D809A/D813A variant (B) of MBP‐MpARF2‐mNG. V_0_ = ~ 170 mL, CV = ~ 530 mL. Monomeric protein elutes around 280 mL. Inner tick marks on the x‐axis indicate the starting points of fractions. (C, D) UV–Vis chromatograms of SD200‐I SEC runs of the K760S variant (C) and the D809A/D813A variant (D) of MpARF2‐mNG. Monomeric protein elutes around 10.5 mL. V_0_ = ~ 7 mL, CV = ~ 23 mL. Inner tick marks on the x‐axis indicate the starting points of fractions. In C, D, only even tick marks are labelled. W = waste. Note that y‐axis scales differ between graphs A–D. (E, F) SDS/PAGE gels highlighting purity of PB1 variants after purification (E) and preparation procedures (F). A_El_, amylose column eluate; M, molecular weight marker. (G) Confocal microscopy images of concentrated monomeric MpARF2‐mNG variants just prior to experiments, highlighting protein homogeneity. MpARF2^K760S^‐mNG concentration is approximately 6.91 μm, MpARF2^D809A/D813A^‐mNG concentration is approximately 3.29 μm. Scale bars 5 μm. A, B and E are representative data from *n* = 2 purifications, C, D, F and G are representative data from *n* = 4 experiments.

During purification, MBP‐MpARF2^K760S^‐mNG and MBP‐MpARF2^D809A/D813A^‐mNG yield a similar SEC profile as for MBP‐MpARF2‐mNG (Fig. 4A,B, compare with Fig. 2B). The composition of the five peaks is also nearly identical between MpARF2 and the PB1 variants (Fig. S4A,C, compare with Fig. 2C). MBP‐MpARF2^K760S^‐mNG yields more monomeric protein than MBP‐MpARF2‐mNG (Fig. 4A, peak c, compare with Fig. 2B), indicating that the yield of monomeric MBP‐MpARF2‐mNG after Ni‐IMAC may be tied to the PB1‐mediated self‐interaction capacity of the protein. However, this does not translate into a higher protein yield after complete purification, as the final yield of MBP‐MpARF2^K760S^‐mNG is comparable to that of MBP‐MpARF2, which is caused by the poor binding of MBP‐MpARF2^K760S^‐mNG to the amylose column (Fig. S4B, A_FT_, Table 2). Although the D809A/D813A mutation also disrupts PB1 multimerisation, we note that MBP‐MpARF2^D809A/D813A^‐mNG yields less monomeric protein compared with MBP‐MpARF2‐mNG and MBP‐MpARF2^K760S^‐mNG (Fig. 4B, peak c, compare Fig. 2B). This observation suggests that PB1 multimerisation is not the only factor affecting the yield of monomeric MBP‐MpARF2‐mNG.

Purification of MBP‐MpARF2^K760S^‐mNG yields 0.89 ± 0.21 mg protein per litre of cell culture and MBP‐MpARF2^D809A/D813A^‐mNG yields 0.14 ± 0.03 mg protein per litre of cell culture (Table 2), both with good purity (Fig. 4E).

After purification, the PB1 variants of MpARF2‐mNG are prepared for experiments in the same way as MpARF2‐mNG. After proteolytic cleavage of the MBP tag and the subsequent analytical SEC step, we note that, like the purification of the MBP‐tagged variants, the yield of monomeric MpARF2^K760S^‐mNG is higher than that of MpARF2‐mNG, and the yield of monomeric MpARF2^D809A/D813A^‐mNG is lower than that of MpARF2‐mNG (Fig. 4C,D, peaks b, compare with Fig. 3A). Monomeric MpARF2^D809A/D813A^‐mNG is poorly resolved from the nucleic‐acid‐bound multimeric species on the SD200‐I column compared with MpARF2‐mNG and MpARF2^K760S^‐mNG (Fig. 4D, compare with Figs 3B and 4C). Nevertheless, the final samples of both MpARF2^K760S^‐mNG and MpARF2^D809A/D813A^‐mNG are pure (Fig. 4F), no micron‐sized structures are detected by microscopy (Fig. 4G, compare with Fig. 3C), and no nucleic‐acid contaminant is detected by UV spectrophotometry.

### The MR of MpARF2—Purification and preparation for experiments

Because the MR of MpARF2 is predicted to be intrinsically disordered [40] and because intrinsically disordered regions (IDRs) are often involved in phase separation [5, 41, 42], we produce this domain (i.e. MpARF2‐MR) in *E. coli*, purify it, and prepare it for experiments to enable future investigation of its phase‐separating properties.

**Fig. 5:**
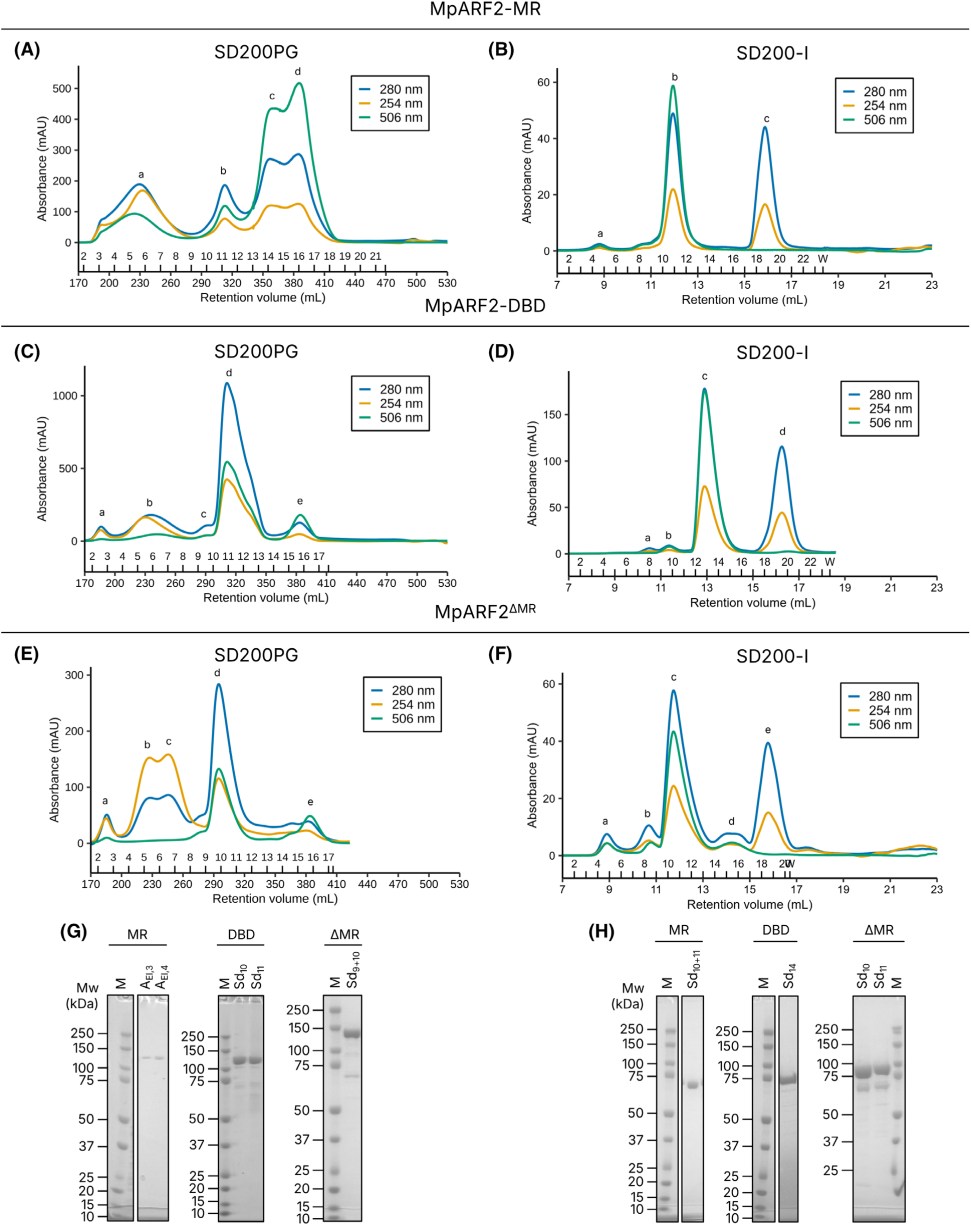
Purification and preparation of MpARF2 truncations. (A, C, E) UV–Vis chromatograms of SD200PG size exclusion chromatography (SEC) runs of maltose‐binding protein (MBP)‐ and mNG‐tagged MpARF2‐MR (A), MpARF2‐DBD (C) and MpARF2^ΔMR^ (E). (B, D, F) UV–Vis chromatograms of SD200‐I SEC runs of mNG‐tagged MpARF2‐MR (B), MpARF2‐DBD (D) and MpARF2^ΔMR^ (F). Inner tick marks indicate starting points of fractions. In B, D, F, only even fractions are labelled, W = waste. Note that y‐axis scales differ between all graphs. MBP‐MpARF2‐MR‐mNG monomer elutes around 310 mL (A), MpARF2‐MR‐mNG monomer elutes around 12 mL (B). MBP‐MpARF2‐DBD‐mNG monomer elutes around 320 mL (C), MpARF2‐DBD‐mNG monomer elutes around 13 mL (D). MBP‐MpARF2^ΔMR^‐mNG monomer elutes around 300 mL (E), MpARF2^ΔMR^‐mNG monomer elutes around 12 mL (F). (G, H) SDS/PAGE analysis of MBP‐MpARF2‐mNG variants after purification (G) and of MpARF2‐mNG variants after preparation for experiments (H). M: molecular weight marker, A_El_: amylose column eluate, Sd: fraction from the SD200PG (G) or the SD200‐I SEC column (H). A is representative from *n* = 5 purifications, B is representative from *n* = 10 purifications, C and F are representative from *n* = 2 purifications, D and E represent a single experiment. The panels in G and H are representative data from the corresponding proteins.

Interestingly, after Ni‐IMAC of MBP‐MpARF2‐MR‐mNG, we again observe a multimeric species co‐eluting with nucleic acids on the SD200PG SEC column (Fig. 5A, peak a). As with MBP‐MpARF2‐mNG, we discard the fractions belonging to this peak. We also discard fractions corresponding to peaks c/d. Despite their high A_506_, these fractions contain little to no MBP‐MpARF2‐MR‐mNG, indicating that they contain degradation products (Fig. S5A). Like MBP‐MpARF2‐mNG, most of the MBP‐MpARF2‐MR‐mNG molecules are cleaved by proteases, as peaks c and d represent the majority of the total A_506_.

Monomeric MBP‐MpARF2‐MR‐mNG elutes around 320 mL on the SD200PG column (Fig. 5A, peak b). Passing the fractions corresponding to this peak over an amylose column reveals that, as is the case for MBP‐MpARF2‐mNG and its PB1 variants, MBP‐MpARF2‐MR‐mNG also binds poorly to this column (Fig. S5B). Nevertheless, a relatively high protein yield is still achieved, namely 2.76 ± 0.20 mg per litre of cell culture (Table 2), with good purity (Fig. 5G).

During protein preparation of MBP‐MpARF2‐MR‐mNG, we again notice secondary cleavage (Fig. S2B), indicating that the secondary cleavage site observed during MBP‐MpARF2‐mNG protein preparation is likely located in the MR. Indeed, the molecular weights of the MpARF2‐MR‐mNG secondary cleavage products and of some MpARF2‐mNG secondary cleavage products correspond to a Q/GP site in the MR (Fig. S2D), the residues that 3C protease cleaves in its canonical LEVLFQ/GP recognition site [43].

After proteolytic removal of the MBP tag, MpARF2‐MR‐mNG solutions remain clear at all concentrations tested up to 20 μm (data not shown). During the subsequent SD200‐I SEC run, monomeric MpARF2‐MR‐mNG elutes at 12 mL (Fig. 5B, peak b) with good purity (Fig. 5H).

### MpARF2‐DBD—Purification and preparation for experiments

Although MpARF2‐DBD has previously been purified using a Chitin‐affinity‐based protocol [30, 38], we adhere to the protocol described here for parity in experiments.

We note that after Ni‐IMAC, MBP‐MpARF2‐DBD‐mNG also retains nucleic acids, as a multimeric species co‐eluting with nucleic acids is observed (Fig. 5C, peak b). The yield of the monomeric MBP‐MpARF2‐DBD‐mNG is so high, with sufficient purity, that further purification via amylose affinity chromatography is optional (Fig. 5C, peak d, Fig. S6A). MBP‐MpARF2‐DBD‐mNG binds poorly to amylose resin (Fig. S6B, A_FT_), resulting in low yields (Table 2). Therefore, we recommend skipping the amylose affinity step.

After isolating MBP‐MpARF2‐DBD‐mNG monomer from the SD200PG, storage and preparation for experiments is performed as for MBP‐MpARF2‐mNG. No premature phase separation is observed during 3C cleavage (data not shown), and the MpARF2‐DBD‐mNG is pure (Fig. 5H).

### MpARF2^ΔMR^ —Purification and preparation for experiments

Just as studying the isolated MR of MpARF2 provides insight into its properties, so too will removing the MR from MpARF2 (i.e. MpARF2^ΔMR^), with the DBD and PB1 domains now covalently linked.

Similar to MBP‐MpARF2‐DBD‐mNG, the purity of monomeric MBP‐MpARF2^ΔMR^‐mNG after Ni‐IMAC followed by SEC also suffices, except for a minor impurity with a molecular weight of ~ 70 kDa (Fig. 5E,G, peak d). MBP‐MpARF2^ΔMR^‐mNG yields so little protein after amylose affinity column chromatography that we skip this purification step (Table 2).

During the proteolytic cleavage step to remove the MBP tag, 3C protease cleaves MBP‐MpARF2^ΔMR^‐mNG poorly, with only slightly more than 50% cleavage after 3.5 h of incubation time (Fig. S2C). To address this, we increased the 3C protease concentration 10‐fold to 10 μg 3C protease per 1 nmol MBP‐MpARF2^ΔMR^‐mNG and incubated for 2 h, which leads to satisfactory cleavage (Fig. S7, first lane). Unlike MpARF2 or MpARF2‐MR, no secondary cleavage is observed, supporting that any secondary 3C sites are in the MR.

The ~ 70 kDa contaminant remains present after the preparation procedure (Fig. 5H). Its consistent presence in Fraction 9 of the SD200PG step across all purifications suggests that this contaminant arises from the Ni‐purification step (Fig. 2B, Figs S4A,C, S5A and S6A).

### MpARF1—Purification of its MR

Our purification protocol has also been successfully applied to purify the MR of MpARF1 (Fig. S8A). The behaviour of MBP‐MpARF1‐MR‐mNG deviates slightly from that of MBP‐MpARF2‐MR‐mNG, in that after passing the Ni eluate over an SD200PG column, the presence of a nucleic‐acid‐bound multimeric species is much lower (Fig. S8B). As with MBP‐MpARF2‐MR‐mNG and other MBP‐MpARF2‐mNG variants, MBP‐MpARF1‐MR‐mNG also binds poorly to the amylose column material, indicating that this issue is not limited to MpARF2 variants (Fig. S8C, A_FT_). After purification, we obtained 1.42 mg of purified MBP‐MpARF1‐MR‐mNG per litre of cell culture (Table 2).

## Discussion

Phase‐separating proteins are an interesting field in cell biology, because they enable the formation of MLOs/biomolecular condensates. To investigate which properties these proteins confer on the condensate and how they influence condensate functions, *in vitro* studies are combined with *in vivo* studies. *In vitro* studies require careful documentation and monitoring of the purification and preparation procedures of the protein of interest, as phase‐separating proteins are often challenging to purify and are very sensitive to seemingly small variations in conditions.

In this study, we describe purification and preparation protocols for MpARF2‐mNG and its variants/truncations. We encountered several challenges, such as protein aggregation and premature phase separation, copurification of degradation products, loss of protein yields due to protein interaction with column materials, and poor reproducibility of the results. The presented protocol overcomes these challenges and enables reproducible purification and preparation of MpARF2‐mNG and its variants for *in vitro* phase separation experiments.

The challenges associated with phase‐separating protein production in *E. coli* also apply to MpARF2. For example, MBP‐MpARF2‐mNG is highly prone to aggregation, resulting in a significant amount of the protein ending up in higher‐order complexes (Fig. 2B).

We note that the affinity of all MBP‐MpARF2‐mNG variants for amylose is low. Although the binding of MBP‐MpARF2‐mNG to amylose is improved by properly reducing the protein's disulfide bonds, a large amount of MBP‐MpARF2‐mNG remains in the amylose column flow‐through. Since all protein variants exhibit poor amylose binding, it is unlikely that any one of the protein domains is the culprit. However, it is possible that the DBD and the MR cause the MBP tag to bind poorly, for example by shielding the amylose binding site of the MBP tag. Another possibility is that the components of the purification buffer cause poor binding of the MBP tag to the amylose resin, as both the glycerol concentration (10%) and the DTT concentration used (5 mm) can reduce the yield on the MBPTrap™ HP column we used [44]. This is supported by the observation that MBP‐MpARF1‐MR‐mNG also binds poorly to the amylose column (Fig. S8C).

We also observe a striking loss in protein yield after removing the MBP tag from MBP‐MpARF2‐mNG and isolating the MpARF2‐mNG monomer: as much as 75% of the protein is lost when using protein concentration filters. Interestingly, the yield loss is only observed with wild‐type MpARF2‐mNG and not with the tested variants (Table 3). This observation strongly suggests that this loss of MpARF2‐mNG is not caused by an interaction of one of its protein domains with the filter material, but, for example, by issues with the solubility of MpARF2‐mNG or by its premature phase separation. Since developing the purification procedure, we have determined *c*_sat_ of MpARF2‐mNG to be around 30 nm under physiological‐like salt conditions [32], showing that MpARF2‐mNG is indeed highly phase‐separation prone.

In summary, despite the reported difficulties with *E. coli* as an expression system to produce phase‐separating proteins [18], we can purify MBP‐MpARF2‐mNG from Rosetta (DE3) *E. coli* in a soluble and monomeric state.

The MR of MpARF2 is predicted to be intrinsically disordered [40]. We note that MpARF2 is strongly targeted by *E. coli* proteases. The Ni‐IMAC purification step of MBP‐MpARF2‐mNG and its variants containing the MR yields a large amount of degradation products, indicating proteolytic cleavage within the MR (Fig. 2B,C, Fig. 5A, Fig. S5A). Indeed, the MpARF2‐DBD and MpARF2^ΔMR^ variants, both of which lack the MR, show relatively little proteolytic degradation (Fig. 5C,E, Fig. S6A). Intrinsically disordered proteins (IDPs) and IDRs are often unstable and present easy targets for proteases [45]. The observation of strong proteolytic cleavage of the MR of MpARF2 by E. *coli* proteases thus supports the predicted intrinsic disorder of this region.

We obtain a relatively low yield of MBP‐MpARF2‐mNG after the SEC step, which is partially due to strong self‐association of the protein. Therefore, we expected that mutations in the PB1 domain that diminish self‐association would improve protein yield. Indeed, MBP‐MpARF2^K760S^‐mNG, in which the invariant lysine is replaced by serine, yields more monomeric protein after SEC than MBP‐MpARF2‐mNG. In contrast, MBP‐MpARF2^D809A/D813A^‐mNG, the yield of which should be similarly affected, yields less monomeric protein after SEC. One explanation could be that the D809A/D813A mutation, which was chosen after previous studies on the AtARF7 PB1 domain [27], may not be sufficient to prevent multimerisation of the MpARF2 PB1 domain.

## Conclusion

MpARF2 and its variants/truncations can be purified in sufficient quantities and prepared in a soluble and monomeric state to enable future reproducible phase separation experiments. This paves the way for elucidating the phase‐separating properties of MpARF2 and the potential functional role of its assemblies in *Marchantia polymorpha* and facilitates similar studies of other ARFs.

## Conflict of interest

The authors declare no conflict of interest.

## Author contributions

BJ developed the protein purification and preparation protocols. BJ and RR carried out the protein purifications. BJ performed the confocal microscopy. SL performed the SEC‐MALS and corresponding analysis. WAMB provided training and essential discussions on the purifications. JWB and CPMM supervised BJ on the experimental work. BJ, JWB and CPMM wrote the manuscript. All authors reviewed the manuscript.

## Supporting information


**Table S1.** Oligos used during cloning/EMSA.
**Table S2.** Source sequences of cloning components.
**Fig. S1.** Validation of the A_506_‐based calculation of protein concentration.
**Fig. S2.** 3C cleavage of MBP‐MpARF2‐mNG, MBP‐MpARF2‐MR‐mNG and MBP‐MpARF2^ΔMR^‐mNG, highlighting secondary cleavage in the MR.
**Fig. S3.** EMSA of MpARF2‐DBD and MBP‐MpARF2‐mNG highlighting that both proteins bind to DNA.
**Fig. S4.** SDS/PAGE gels of samples from the purifications of MBP‐MpARF2^K760S^‐mNG and MBP‐MpARF2^D809A/D813A^‐mNG, respectively.
**Fig. S5.** SDS/PAGE gels of samples from the purification and preparation of (MBP‐)MpARF2‐MR‐mNG.
**Fig. S6.** SDS/PAGE gels of samples from the purification of MBP‐MpARF2‐DBD‐mNG.
**Fig. S7.** SDS/PAGE gel of samples from the SD200‐I run of MpARF2^ΔMR^‐mNG.
**Fig. S8.** Purification of MpARF1‐MR.

## Data Availability

Data will be made available upon request.

## References

[1] Lafontaine DLJ , Riback JA , Bascetin R and Brangwynne CP (2020) The nucleolus as a multiphase liquid condensate. Nat Rev Mol Cell Biol 22, 165–182. 32873929 10.1038/s41580-020-0272-6

[2] Love AJ , Yu C , Petukhova NV , Kalinina NO , Chen J and Taliansky ME (2017) Cajal bodies and their role in plant stress and disease responses. RNA Biol 14, 779–790. 27726481 10.1080/15476286.2016.1243650 PMC5519230

[3] Yang P , Mathieu C , Kolaitis RM , Zhang P , Messing J , Yurtsever U , Yang Z , Wu J , Li Y , Pan Q *et al*. (2020) G3BP1 is a tunable switch that triggers phase separation to assemble stress granules. Cell 181, 325–345. 32302571 10.1016/j.cell.2020.03.046 PMC7448383

[4] Brangwynne CP , Eckmann CR , Courson DS , Rybarska A , Hoege C , Gharakhani J , Jülicher F and Hyman AA (2009) Germline P granules are liquid droplets that localize by controlled dissolution/condensation. Science 324, 1729–1732. 19460965 10.1126/science.1172046

[5] Banani SF , Lee HO , Hyman AA and Rosen MK (2017) Biomolecular condensates: organizers of cellular biochemistry. Nat Rev Mol Cell Biol 18, 285–298. 28225081 10.1038/nrm.2017.7 PMC7434221

[6] Alberti S , Gladfelter A and Mittag T (2019) Considerations and challenges in studying liquid‐liquid phase separation and biomolecular condensates. Cell 176, 419–434. 30682370 10.1016/j.cell.2018.12.035 PMC6445271

[7] Emenecker RJ , Holehouse AS and Strader LC (2021) Biological phase separation and biomolecular condensates in plants. Annu Rev Plant Biol 72, 17–46. 33684296 10.1146/annurev-arplant-081720-015238 PMC8221409

[8] Ruff KM , Roberts S , Chilkoti A and Pappu RV (2018) Advances in understanding stimulus‐responsive phase behavior of intrinsically disordered protein polymers. J Mol Biol 430, 4619–4635. 29949750 10.1016/j.jmb.2018.06.031

[9] Wang J , Choi JM , Holehouse AS , Lee HO , Zhang X , Jahnel M , Maharana S , Lemaitre R , Pozniakovsky A , Drechsel D *et al*. (2018) A molecular grammar governing the driving forces for phase separation of prion‐like RNA binding proteins. Cell 174, 688–699. 29961577 10.1016/j.cell.2018.06.006 PMC6063760

[10] Martin EW , Holehouse AS , Peran I , Farag M , Incicco JJ , Bremer A , Grace CR , Soranno A , Pappu RV and Mittag T (2020) Valence and patterning of aromatic residues determine the phase behavior of prion‐like domains. Science 367, 694–699. 32029630 10.1126/science.aaw8653 PMC7297187

[11] Boeynaems S , Alberti S , Fawzi NL , Mittag T , Polymenidou M , Rousseau F , Schymkowitz J , Shorter J , Wolozin B , Van Den Bosch L *et al*. (2018) Protein phase separation: a new phase in cell biology. Trends Cell Biol 28, 420–435. 29602697 10.1016/j.tcb.2018.02.004 PMC6034118

[12] Zhang Y , Pyo AG , Kliegman R , Jiang Y , Brangwynne CP , Stone HA and Wingreen NS (2024) The exchange dynamics of biomolecular condensates. elife 12, RP91680. 39320949 10.7554/eLife.91680 PMC11424094

[13] Banani SF , Rice AM , Peeples WB , Lin Y , Jain S , Parker R and Rosen MK (2016) Compositional control of phase‐separated cellular bodies. Cell 166, 651–663. 27374333 10.1016/j.cell.2016.06.010 PMC4967043

[14] Dorone Y , Boeynaems S , Flores E , Jin B , Hateley S , Bossi F , Lazarus E , Pennington JG , Michiels E , De Decker M *et al*. (2021) A prion‐like protein regulator of seed germination undergoes hydration‐dependent phase separation. Cell 184, 4284–4298. 34233164 10.1016/j.cell.2021.06.009 PMC8513799

[15] Szczerba M , Johnson B , Acciai F , Gogerty C , McCaughan M , Williams J , Kibler KV and Jacobs BL (2023) Canonical cellular stress granules are required for arsenite‐induced necroptosis mediated by Z‐DNA‐binding protein 1. Sci Signal 16, eabq0837. 36917643 10.1126/scisignal.abq0837 PMC10561663

[16] Linsenmeier M , Hondele M , Grigolato F , Secchi E , Weis K and Arosio P (2022) Dynamic arrest and aging of biomolecular condensates are modulated by low‐complexity domains, RNA and biochemical activity. Nat Commun 13, 3030. 35641495 10.1038/s41467-022-30521-2 PMC9156751

[17] Sundaravadivelu Devarajan D , Wang J , Szała‐Mendyk B , Rekhi S , Nikoubashman A , Kim YC and Mittal J (2024) Sequence‐dependent material properties of biomolecular condensates and their relation to dilute phase conformations. Nat Commun 15, 1–14. 38429263 10.1038/s41467-024-46223-w PMC10907393

[18] Alberti S , Saha S , Woodruff JB , Franzmann TM , Wang J and Hyman AA (2018) A User's guide for phase separation assays with purified proteins. J Mol Biol 430, 4806–4820. 29944854 10.1016/j.jmb.2018.06.038 PMC6215329

[19] Ceballos AV , McDonald CJ and Elbaum‐Garfinkle S (2018) Methods and strategies to quantify phase separation of disordered proteins. Methods Enzymol 611, 31–50. 30471691 10.1016/bs.mie.2018.09.037 PMC6688841

[20] Graether SP (2019) Troubleshooting guide to expressing intrinsically disordered proteins for use in NMR experiments. Front Mol Biosci 5, 427532. 10.3389/fmolb.2018.00118 PMC6345686 30713842

[21] Kar M , Dar F , Welsh TJ , Vogel LT , Kühnemuth R , Majumdar A , Krainer G , Franzmann TM , Alberti S , Seidel CAM *et al*. (2022) Phase‐separating RNA‐binding proteins form heterogeneous distributions of clusters in subsaturated solutions. Proc Natl Acad Sci USA 119, e2202222119. 35787038 10.1073/pnas.2202222119 PMC9282234

[22] Das S , Weijers D and Borst JW (2021) Auxin response by the numbers. Trends Plant Sci 26, 442–451. 33500193 10.1016/j.tplants.2020.12.017

[23] Tan X , Calderon‐Villalobos LIA , Sharon M , Zheng C , Robinson CV , Estelle M and Zheng N (2007) Mechanism of auxin perception by the TIR1 ubiquitin ligase. Nature 446, 640–645. 17410169 10.1038/nature05731

[24] Tiwari SB , Wang X‐J , Hagen G and Guilfoyle TJ (2001) AUX/IAA proteins are active repressors, and their stability and activity are modulated by auxin. Plant Cell 13, 2809–2822. 11752389 10.1105/tpc.010289 PMC139490

[25] Tiwari SB , Hagen G and Guilfoyle T (2003) The roles of auxin response factor domains in auxin‐responsive transcription. Plant Cell 15, 533–543. 12566590 10.1105/tpc.008417 PMC141219

[26] Boer DR , Freire‐Rios A , Van Den Berg WAM , Saaki T , Manfield IW , Kepinski S , López‐Vidrieo I , Franco‐Zorrilla JM , De Vries SC , Solano R *et al*. (2014) Structural basis for DNA binding specificity by the auxin‐dependent ARF transcription factors. Cell 156, 577–589. 24485461 10.1016/j.cell.2013.12.027

[27] Korasick DA , Westfall CS , Lee SG , Nanao MH , Dumas R , Hagen G , Guilfoyle TJ , Jez JM and Strader LC (2014) Molecular basis for AUXIN RESPONSE FACTOR protein interaction and the control of auxin response repression. Proc Natl Acad Sci USA 111, 5427–5432. 24706860 10.1073/pnas.1400074111 PMC3986151

[28] Nanao MH , Vinos‐Poyo T , Brunoud G , Thévenon E , Mazzoleni M , Mast D , Lainé S , Wang S , Hagen G , Li H *et al*. (2014) Structural basis for oligomerization of auxin transcriptional regulators. Nat Commun 5, 1–8. 10.1038/ncomms4617 24710426

[29] Ulmasov T , Hagen G and Guilfoyle TJ (1999) Activation and repression of transcription by auxin‐response factors. Proc Natl Acad Sci USA 96, 5844–5849. 10318972 10.1073/pnas.96.10.5844 PMC21948

[30] Kato H , Mutte SK , Suzuki H , Crespo I , Das S , Radoeva T , Fontana M , Yoshitake Y , Hainiwa E , van den Berg W *et al*. (2020) Design principles of a minimal auxin response system. Nat Plants 6, 473–482. 32415296 10.1038/s41477-020-0662-y

[31] Mutte SK , Kato H , Rothfels C , Melkonian M , Wong GKS and Weijers D (2018) Origin and evolution of the nuclear auxin response system. elife 7, e33399. 29580381 10.7554/eLife.33399 PMC5873896

[32] Arfman K , Janssen BPJ , Romein R , van den Boom S , van der Woude M , Jansen L , Rademaker M , Hernández‐García J , Ramalho JJ , Dipp‐Álvarez M *et al*. (2025) Nanoclustering of a plant transcription factor enables strong yet specific DNA binding. *bioRxiv* 2025.11.05.686732. 10.1126/sciadv.aed7904 PMC13398500 42497281

[33] Fontana M , Roosjen M , García IC , van den Berg W , Malfois M , Boer R , Weijers D and Hohlbein J (2023) Cooperative action of separate interaction domains promotes high‐affinity DNA binding of Arabidopsis thaliana ARF transcription factors. Proc Natl Acad Sci USA 120, e2219916120. 36881630 10.1073/pnas.2219916120 PMC10089223

[34] Das S , de Roij M , Bellows S , Kohlen W , Farcot E , Weijers D and Borst JW (2022) Selective degradation of ARF monomers controls auxin response in Marchantia. *bioRxiv* 2022.11.04.515187.

[35] Shaner NC , Lambert GG , Chammas A , Ni Y , Cranfill PJ , Baird MA , Sell BR , Allen JR , Day RN , Israelsson M *et al*. (2013) A bright monomeric green fluorescent protein derived from *Branchiostoma lanceolatum* . Nat Methods 10, 407–409. 23524392 10.1038/nmeth.2413 PMC3811051

[36] Some D , Amartely H , Tsadok A and Lebendiker M (2019) Characterization of proteins by size‐exclusion chromatography coupled to multi‐angle light scattering (SEC‐MALS). J Vis Exp e59615. 10.3791/59615 31282880

[37] Hernández‐García J , Carrillo‐Carrasco VP , Rienstra J , Tanaka K , de Roij M , Dipp‐Álvarez M , Freire‐Ríos A , Crespo I , Boer R , van den Berg WAM *et al*. (2024) Evolutionary origins and functional diversification of auxin response factors. Nat Commun 15, 10909. 39738167 10.1038/s41467-024-55278-8 PMC11685440

[38] Crespo I , Malfois M , Rienstra J , Tarrés‐Solé A , van den Berg W , Weijers D and Boer DR (2025) The structure and function of the DNA binding domain of class B MpARF2 share more traits with class a AtARF5 than to that of class B AtARF1. Structure 33, 960–973. 40086441 10.1016/j.str.2025.02.006

[39] Novy R and Morris B (2001) Use of glucose to control basal expression in the pET system. Innovations 13, 8–10.

[40] Roosjen M , Paque S and Weijers D (2018) Auxin response factors: output control in auxin biology. J Exp Bot 69, 179–188. 28992135 10.1093/jxb/erx237

[41] Martin EW and Holehouse AS (2020) Intrinsically disordered protein regions and phase separation: sequence determinants of assembly or lack thereof. Emerg Top Life Sci 4, 307–329. 10.1042/ETLS20190164 33078839

[42] Ibrahim AY , Khaodeuanepheng NP , Amarasekara DL , Correia JJ , Lewis KA , Fitzkee NC , Hough LE and Whitten ST (2023) Intrinsically disordered regions that drive phase separation form a robustly distinct protein class. J Biol Chem 299, 102801. 36528065 10.1016/j.jbc.2022.102801 PMC9860499

[43] Fan X , Li X , Zhou Y , Mei M , Liu P , Zhao J , Peng W , Jiang ZB , Yang S , Iverson BL *et al*. (2020) Quantitative analysis of the substrate specificity of human rhinovirus 3C protease and exploration of its substrate recognition mechanisms. ACS Chem Biol 15, 63–73. 31613083 10.1021/acschembio.9b00539

[44] Cytiva (2020) MBPTrap™ HP 1 mL and 5 mL Instructions for Use.

[45] Paz A , Zeev‐Ben‐Mordehai T , Sussman JL and Silman I (2010) Purification of intrinsically disordered proteins. In Instrumental Analysis of Intrinsically Disordered Proteins: Assessing Structure and Conformation ( Uversky V and Longhi S , eds), pp. 695–704. John Wiley & Sons, Hoboken, NJ.

[46] Kozlowski LP (2016) IPC ‐ Isoelectric Point Calculator. Biol Direct 11, 1–16. 27769290 10.1186/s13062-016-0159-9 PMC5075173

