# Supplementary material for: Purification and preparation of Marchantia polymorpha Auxin Response Factor 2 for phase separation studies
Source: FEBS Open Bio. 2026 Jul 21:10.1002/2211-5463.70313. Online ahead of print. doi: 10.1002/2211-5463.70313 (PMC13398774; doi:10.1002/2211-5463.70313)
Supplement: Supplementary file 1 — Table S1. Oligos used during cloning/EMSA. Table S2. Source sequences of cloning components. Fig. S1. Validation of the A506‐based calculation of protein concentration. Fig. S2. 3C cleavage of MBP‐MpARF2‐mNG, MBP‐MpARF2‐MR‐mNG and MBP‐MpARF2ΔMR‐mNG, highlighting secondary cleavage in the MR. Fig. S3. EMSA of MpARF2‐DBD and MBP‐MpARF2‐mNG highlighting that both proteins bind to DNA. Fig. S4. SDS/PAGE gels of samples from the purifications of MBP‐MpARF2K760S‐mNG and MBP‐MpARF2D809A/D813A‐mNG, respectively. Fig. S5. SDS/PAGE gels of samples from the purification and preparation of (MBP‐)MpARF2‐MR‐mNG. Fig. S6. SDS/PAGE gels of samples from the purification of MBP‐MpARF2‐DBD‐mNG. Fig. S7. SDS/PAGE gel of samples from the SD200‐I run of MpARF2ΔMR‐mNG. Fig. S8. Purification of MpARF1‐MR. [file FEB4-9999-0-s001.docx]

**Table S1. Oligos used during cloning/EMSA.** Oligos are ssDNA unless mentioned otherwise.

| Oligo | Sequence (5’-3’) |
| --- | --- |
| MCS dsDNA oligo | GAGGTATTATTTCAAGGACCGGGATCCGAATTCGAGCGCCGTCGACAAGCTTGCGGCCGCACTGCAGGAAAACCTGTACTTCCAAT |
| BPJ002 | TCCTTGAAATAATACCTCTAGCTCGATCCCATTAGTCTGCG |
| BPJ005 | AAAACCTGTACTTCCAATCGGACGAGATGGTGAGCAAGGGCGA |
| BPJ013 | gaattcTCTCCCTCCACGACCATCA |
| BPJ014 | aagcttATTGGATCTCTGGGGACCC |
| BPJ029 | ggttcttctggttcttctatgaaaatcgaagaagg |
| BPJ030 | agaagaaccagaagaacccatggtatatctccttc |
| BPJ031 | aaatcttctggtcaccatcaccatcaccattgagatccggctgctaac |
| BPJ032 | atggtgatggtgatggtgaccagaagatttCTTGTACAGCTCGTCCATGC |
| BPJ043 | AAGCTTCATGTCGTCGCCGCGA |
| BPJ061 | GACGTCTTGAAGGGCTCCACTTCCCATGGT |
| BPJ062 | GAGCCCTTCAAGACGTCGCAAAATTTCCAACAAG |
| BPJ121 | ttcaaggaccgggatccgaattcacaacccctttcctgatct |
| BPJ122 | tcctgcagtgcggccgcaagcttctgcataaattggctatcatttatac |
| BPJ161 | AAGCTTGCGGCCGCACT |
| BPJ162 | TTCGGATCCCGGTCCTTGAAAT |
| BPJ163 | CAAGGACCGGGATCCGAAATGTCAGAAGCA |
| BPJ164 | CAGTGCGGCCGCAAGcttATTGGATCTCTGGGGACCCTTATC |
| BPJ165 | CAGTGCGGCCGCAAGcttGAAGGGCTCCACTTCCCATG |
| BPJ166 | CAGTGCGGCCGCAAGcttCATGTCGTCGCCGCG |
| PC019 | **Cy5**-gatacacgcttgtcggccaaaggccgacaaccactca |
| PC020 | tgagtggttgtcggcctttggccgacaagcgtgtatc |

**Table S2. Source sequences of cloning components**

| MBP | ATGAAAATCGAAGAAGGTAAACTGGTAATCTGGATTAACGGCGATAAAGGCTATAACGGTCTCGCTGAAGTCGGTAAGAAATTCGAGAAAGATACCGGAATTAAAGTCACCGTTGAGCATCCGGATAAACTGGAAGAGAAATTCCCACAGGTTGCGGCAACTGGCGATGGCCCTGACATTATCTTCTGGGCACACGACCGCTTTGGTGGCTACGCTCAATCTGGCCTGTTGGCTGAAATCACCCCGGACAAAGCGTTCCAGGACAAGCTGTATCCGTTTACCTGGGATGCCGTACGTTACAACGGCAAGCTGATTGCTTACCCGATCGCTGTTGAAGCGTTATCGCTGATTTATAACAAAGATCTGCTGCCGAACCCGCCAAAAACCTGGGAAGAGATCCCGGCGCTGGATAAAGAACTGAAAGCGAAAGGTAAGAGCGCGCTGATGTTCAACCTACAAGAACCGTACTTCACCTGGCCGCTGATTGCTGCTGACGGGGGTTATGCGTTCAAGTATGAAAACGGCAAGTACGACATTAAAGACGTGGGCGTGGATAACGCTGGCGCGAAAGCGGGTCTGACCTTCCTGGTTGACCTGATTAAAAACAAACACATGAATGCAGACACCGATTACTCCATCGCAGAAGCTGCCTTTAATAAAGGCGAAACAGCGATGACCATCAACGGCCCGTGGGCATGGTCCAACATCGACACCAGCAAAGTGAATTATGGTGTAACGGTACTGCCGACCTTCAAGGGTCAACCATCCAAACCGTTCGTTGGCGTGCTGAGCGCAGGTATTAACGCCGCCAGTCCGAACAAAGAGCTGGCAAAAGAGTTCCTCGAAAACTATCTGCTGACTGATGAAGGTCTGGAAGCGGTTAATAAAGACAAACCGCTGGGTGCCGTAGCGCTGAAGTCTTACGAGGAAGAGTTGGCGAAAGATCCACGTATTGCCGCCACTATGGAAAACGCCCAGAAAGGTGAAATCATGCCGAACATCCCGCAGATGTCCGCTTTCTGGTATGCCGTGCGTACTGCGGTGATCAACGCCGCCAGCGGTCGTCAGACTGTCGATGAAGCCCTGAAAGACGCGCAGACT |
| --- | --- |
| mNG | ATGGTGAGCAAGGGCGAGGAGGATAACATGGCCTCTCTCCCAGCGACACATGAGTTACACATCTTTGGATCCATCAACGGTGTGGACTTTGACATGGTGGGTCAGGGCACCGGCAATCCAAATGATGGTTATGAGGAGTTAAACCTGAAGTCCACCAAGGGTGACCTCCAGTTCTCCCCCTGGATTCTGGTCCCTCATATCGGGTATGGCTTCCATCAGTACCTGCCCTACCCTGACGGGATGTCGCCTTTCCAGGCCGCCATGGTAGATGGCTCCGGATACCAAGTCCATCGCACAATGCAGTTTGAAGATGGTGCCTCCCTTACTGTTAACTACCGCTACACCTACGAGGGAAGCCACATCAAAGGAGAGGCCCAGGTGAAGGGGACTGGTTTCCCTGCTGACGGTCCTGTGATGACCAACTCGCTGACCGCTGCGGACTGGTGCAGGTCGAAGAAGACTTACCCCAACGACAAAACCATCATCAGTACCTTTAAGTGGAGTTACACCACTGGAAATGGCAAGCGCTACCGGAGCACTGCGCGGACCACCTACACCTTTGCCAAGCCAATGGCGGCTAACTATCTGAAGAACCAGCCGATGTACGTGTTCCGTAAGACGGAGCTCAAGCACTCCAAGACCGAGCTCAACTTCAAGGAGTGGCAAAAGGCCTTTACCGATGTGATGGGCATGGACGAGCTGTACAAG |
| MpARF2-DBD | ATGTCAGAAGCATCTTCCATCACTCGTCACCCCTACAAGGCAAACACCGGGCCCCTGCTGAAGTTCCAGCAATCGTCGGATGCTTCATCCTTGCCGCCAATGGCACGTCCCATGGCGAGCAGACAGCTTGCTACATCTCACACCGCTGCCTCCAACGTCTCCGTAGCAGGCGATGATGGCATCGATGCCGAGCTGTGGTACGCCTGTGCTGGTCCTCAGAAGGCATTACCACCCGTAGGCAGCGTCGTGGCCTACTTGCCTCAAGGTCACATAGAGCAGGTCGCGTCTTTCAATAATCAAGAACTCGACGCCCAAATTCCTCGGTATAATCTGCCCGCAGTGATACCATGTATGCTCAACGACATACAACTCAGCGCGGATCCTGATTCCGACGAGGTCTATGCGACTCTCACCCTGTGTCCCATGAGCGAGCAACACGAAGACTCGTCCGACTGCGCCGAGCCCCCGCCACCTCCCAAGAGGAAGTCCCGCAGTTTTACCAAAACCCTCACTGTCTCTGATACCAGCACGCATGGAGGCTTCTCGGTGCCCCGACGCGCTGCCGATGACTGCTTGCCGAAATTGGATATGAGTCTCAACCCTCCAAATCAGGAATTAGTGGCCAAGGATCTTCATGGCAACGAATGGCGATTTCGTCACATATTTCGAGGTCAACCTAAACGCCACCTTCTCACTACGGGATGGAGCGTATTTGTGAGCCAAAAGAGGCTCGTCGCCGGAGACGCGGTGCTGTTTCTCAGGGGTGAGAATGGCCAGCTCAGAGTAGGAGTCCGGCGTGCTCCTCGCCAGCAACAACTGCAACCCAAAGTGTTGACGTCTCCAACGATGCACATCGGGGTTCTCGCGGCCGCTGCCCATGCGGCAACGGAGAAATCTCGATTCTCTCTAATTTACAACCCTCGATCTTGCCCTTCGGAGTTCGTTATTCCGTACTCTAAGTACCTCAAGGCTGTGAAAAGCAACTTCAACGTTGGCCAGCGTTTTAAAATGAAGTTCGAATCGGAGGACCCTTCAGATAGGAGGCATACGGGAACTATCACCGGAATTTGTGATTTCGACCCTGCCAGGTGGCCTGGCTCAGAGTGGAGATCCCTCCAGGTTAATTGGGACGAATCGTCCTCGAGCGAGAGACAGGAGAGGGTCTCACCATGGGAAGTGGAGCCCTTC |
| MpARF2-MR | TCTCCCTCCACGACCATCACTCCGTCGGTCAGTACTAGAAAGCGATTGAGGCCGGTCACCCAACCACATTCTGAGTCAGTGAACAGGAATGCCGTCGAGACAAGTAAGGCGCAAACTCAAACGATGAGGCTAGCGAGAGCTTTCCACGGCGGTCATGAAATGCTGCCGTCATCAGCCGAGGAGGAGGATGCGGAATCTCTTTCTGCCAAAATGTCTTGGATTAAGAGAGAGGACAACTTCAAGAGCGAAGCGCAGAGCGTTGGCTCTAGACAGGGCCCGGATAGTTGGATGTCTATCAGGAGACCCGACCCAGTGCAGGTCCCTGACATGTTTAGAAATCTCCCGGCTTCGGGAGTGCCGGATCTTCGAGGTATGATTGGCATCGAGAGGCGCCAGCAGGAACACTTAAAGTTCTGCGTCAAGCAATATCGGGAAAACAAGGATGAAATCTCGGGAACTACTCTGCAGCTGTCAAGTCCCCGACCTCCGAACTTGCAGAATTACGTCAAGTCATCCACAGATCTGAATCTCTCCGTGTCCTCGCCTGCGTCGAGCAACAAGGGATCGAGCCTTCTTTGGTCCAATTCTCAGTCTGTAACGTTGCCATCGTATAATGGTCACGAGTCGACGAACGCATCCTCTTGGCTCTCGTTTAGACCGGGACAGAGCGATGTTGCCGCCTCTTCGCCTCACTGCACTACATTGTCAATGAGCAATCTGCCTCCAGCTGATTCTGAAACGTCAAGCCATCCTTCGACTCCGAAGAGTTATCTATGGGAGAAGAGGATAAGGATGGAGCCGGACACGAACCGAGCAGCTGCTCCCGTTCAGAGTGAGCAAAAATGTAAAATTTTCGGGGTGCCTCTCGACAAGCCAACTCCGATCGTGATTCCTTCTCAAGTGCCAGGATCTAAGGCAGTGAGGAGCACGGACGATGGATCAGGACCGAGCAGCAGTCGTGGGCTGGAGAAAGTCGTGTCTCCCAGTCCGACTTCTTCCGCAGTCGGAGGACAGGAGCAGGATAAGGGTCCCCAGAGATCCAAT |
| MpARF2-PB1 | AAGACGTCGCAAAATTTCCAACAAGGTCCAGTTCGTAGCTACACGAAGATTCACAAGCAGGGATCGTTCGGAAGATCGATCGACGTCCAAAGTTATGACGGCTACACCGACTTGTTAAGAAAGGTAGAAAACATGTTCGAGCTGAACGGAGAGCTGTTCGACAAGAAGTCCGGGTGGCAGCTCGTGTACACAGACCACGAAGACGACGTCCTCCTCGTGGGAGACGATCCCTGGATGGAGTTTGTGAGTTGCGTGAGGACGCTGAGACTGCTGAGCCCGGGCGAGGCGTCGTCGTCGGGCAAGAGCGGGCAGTCGCACGACGAGGACGCGGGCGCGGGCAAGGACGGGGCCAAGCGGTGCGACTCGTCCTCGCCGTCGGCGGGGGCTCGCGGCGACGACATG |
| MpARF1-MR | CTGCATAAATTGGCTATCATTTATACTACCATGGGATATTATGCCAGGGCTGATGGGCAGATCTTGTCCCGCCGATGAGCAATAAGGAGCAGGCAGTAGGCTACCGCCAGCAAATCGCGCCTGGTTATCCTTCGCCTTACCAAAGCCCCTTGATCCCAGACCCCCAGCTCCGTTTGGCATTTGATCAATGGATACACCGAAAAGGAGATGACTACGAGGATCTGACTGTACTTGATCTTGCTCCTGTGAATTGTCCCGAAACACTGCATAAGTAGGATTCTGCATGGGTAGGGCTGTCAAACCAGATTGACAAGGTTCATTGGATGAAAGAGCATATGTGGGAGCAGAACTGGGGGATGAGTCTGCTCGCCCCATGCGGGAACTTGGATGAGCGCAATCGGATGTAGTGGAATCCCGCATAGAATACCAGGCACTCGATACAGGAGAGTCCCGCCCTACCATAGCGGAACCAGGTAGCATAGTAGCTTGTTGCATTGCGCTTTGATTGCTGCGGAGAAGATTTGAAAACTGATTTGTATCTTCAGTGGTGAGTACACTGCCTTGTTGAGTCCTGCTCAGAAGACTCTGTAAAGGAAATGATCCTGACGCAGTGGAAGCAGGAGATAAGTGCACATCCGAGTCCGTATATCCTGATGACGCCCGCAATGTAGATAACTGCATAGGAGATTGTGGCCTGCTTGATGAAAGTTGGAGTAAGGGCCCAGTAACATCAGGTAATGGCAACGGCTGAGGAGACTGCAGAACTTGTTGCTGCTGCTGTTGCATATTAAGCTGCTGCTGTTGTTGCTGTTGTTGCTGCTGTTGTTGCTGCTGCTCTTGTTGCTGTAGAGGAGGTTGTTGCTGCTGGGGACGAAATTGCATCTGATGATTCTGCAAGGACTGCTGTGGTTGTCGCTGAAGCAGTTGCTTGGACGTCTCGTTGCCGCGAATCTCTTGAAGAGCAGCCGCAGCCATGACCCGATAAAAATCTGTTGGTGTACCAGGAGCTACTGAATCTGGACTCTGTGGTAACCTGGGTGTCCAAGGGTCCATCCCAAGGCCCCTGAAAGGTAGATTCGATAGACCGTCTCTCTCGTCATCTCTTAACCACATAGAAGACTTCTTGACCGAGCCATCTCCTTCCTCATCTTCACCCGAATGGCCTCGAGAAGACGCCCGGGGCCGCTTCGCTCTCAATGTCAGTGGAGGACAGATCAGGAAAGGGGTTGT |

**
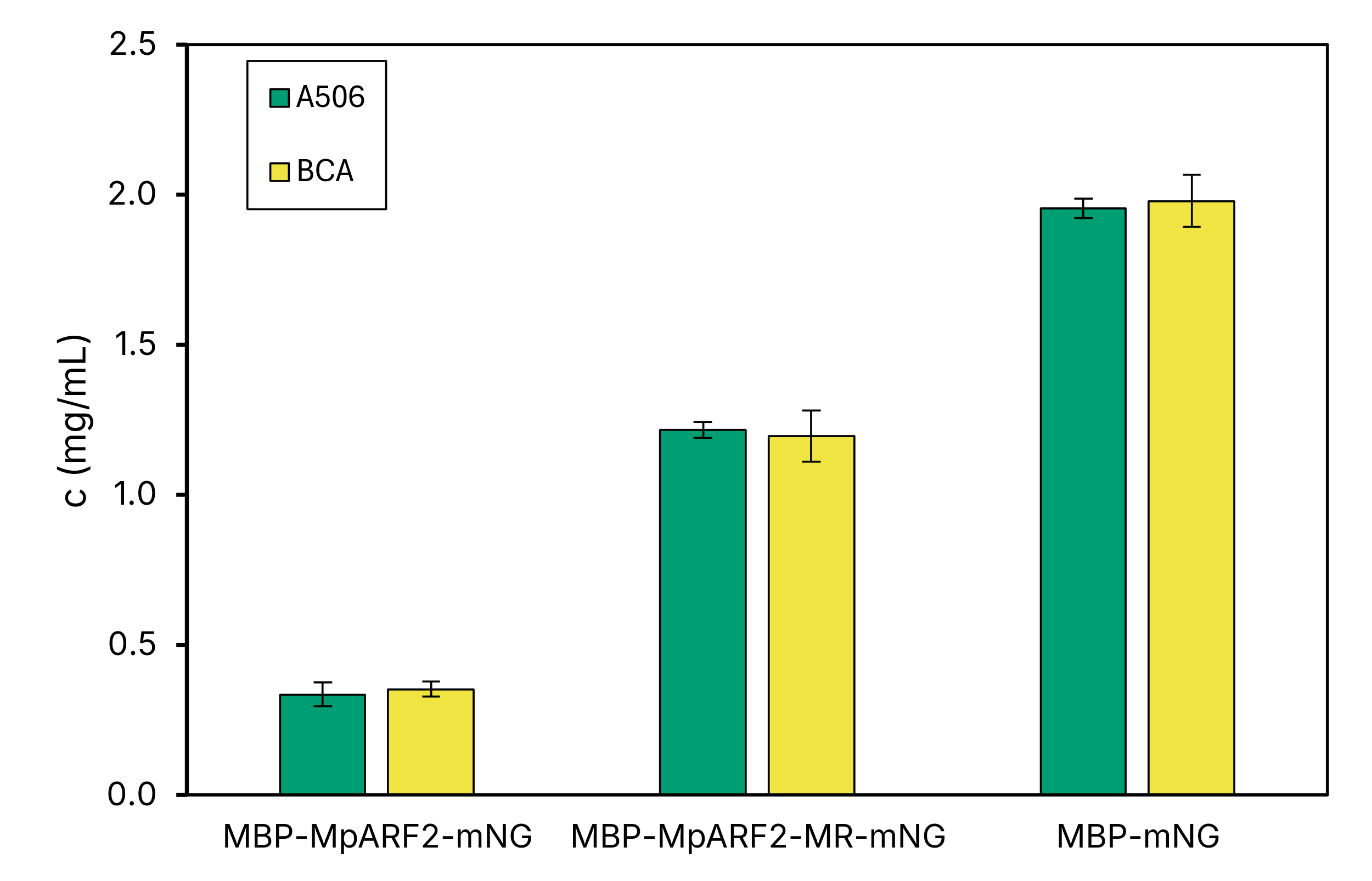
**

**Figure S1. Validation of the A_506_-based calculation of protein concentration.** Mean concentration values of protein samples as calculated by A_506_ (and the ε_506_ of mNG, 116000 M^-1^ cm^-1^) and by BCA assay (n=3 for both assays). Calculating protein concentration based on the A_506_ and the ε_506_ of mNG (116000 M^-1^ cm^-1^) shows excellent agreement with a BCA quantification for three different proteins. Error bars represent the standard deviation.


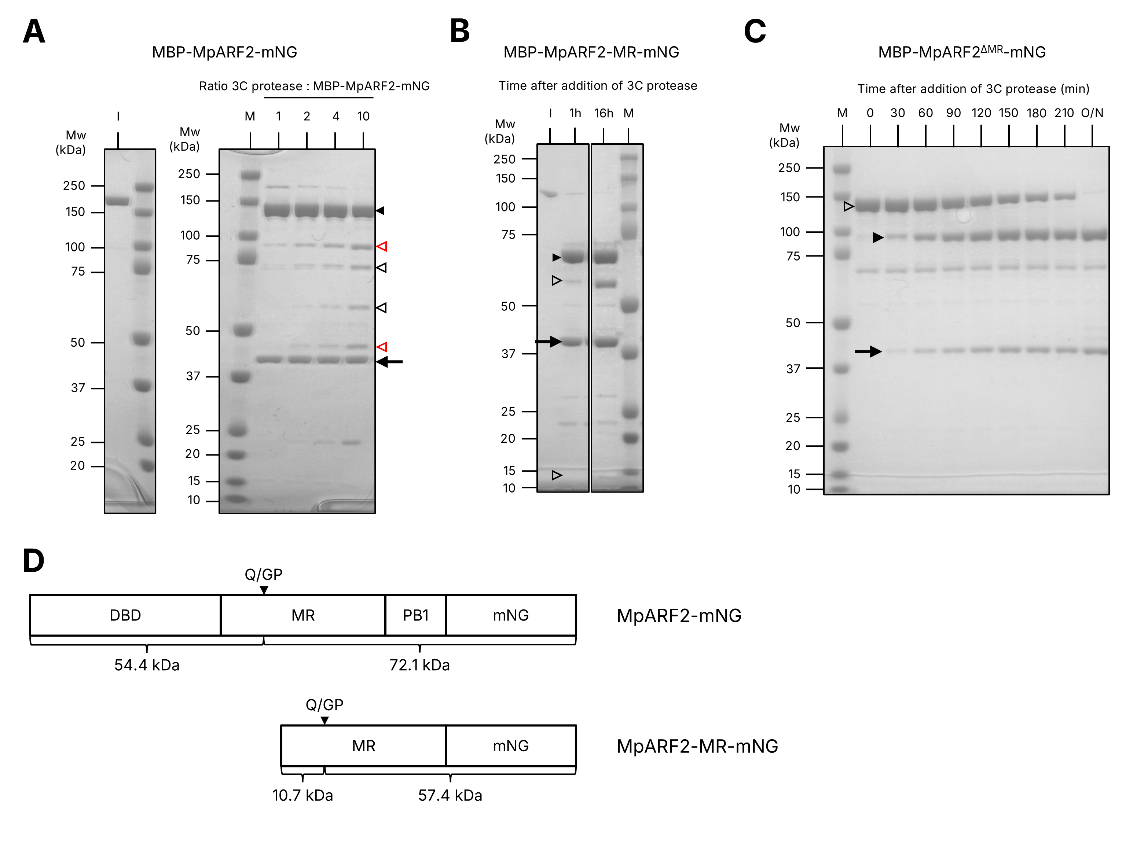


**Figure S2. 3C cleavage of MBP-MpARF2-mNG, MBP-MpARF2-MR-mNG and MBP-MpARF2^ΔMR^-mNG, highlighting secondary cleavage in the MR. (A)** 3C cleavage of MBP-MpARF2-mNG at various ratios of 3C protease to MBP-MpARF2-mNG. Conditions: Amylose elution buffer at RT, incubated for 2h. The ratios above the gel are expressed as μg 3C protease per nmol MBP-MpARF2-mNG. Black arrowheads indicate desired cleavage product. Black open arrowheads indicate secondary cleavage products which are due to the presence of a secondary Q/GP site (see **D**). Red open arrowheads indicate secondary cleavage products arising from a different, unknown cleavage site. Black arrow indicates position of free MBP. **(B)** 3C cleavage of MBP-MpARF2-MR-mNG, highlighting secondary cleavage over time. Conditions: 50 mM Tris pH 7.5, 125 mM NaCl at 4 °C, 1 μg 3C protease per 0.1 nmol MBP-MpARF2-MR-mNG. The black arrowhead indicates the desired cleavage product. Open arrowheads indicate secondary cleavage products. Black arrow indicates free MBP. Note that the 10.7 kDa protein is barely detectable by Coomassie staining. **(C)** 3C cleavage of MBP-MpARF2^ΔMR^-mNG. The numbers above the gel indicate the number of minutes after the addition of 3C protease, O/N = overnight. The open arrowhead indicates MBP-tagged protein, the black arrowhead indicates MBP-free protein of ~88 kDa, the black arrow indicates free MBP. Conditions: Amylose elution buffer at RT, 1 μg 3C protease per 1 nmol MBP-MpARF2^ΔMR^-mNG. **(D)** Position of the secondary Q/GP site in MpARF2-mNG and MpARF2-MR-mNG proteins, with molecular weights of the resulting cleavage products indicated. **(A-C)** I: input, M: Molecular weight marker.

*
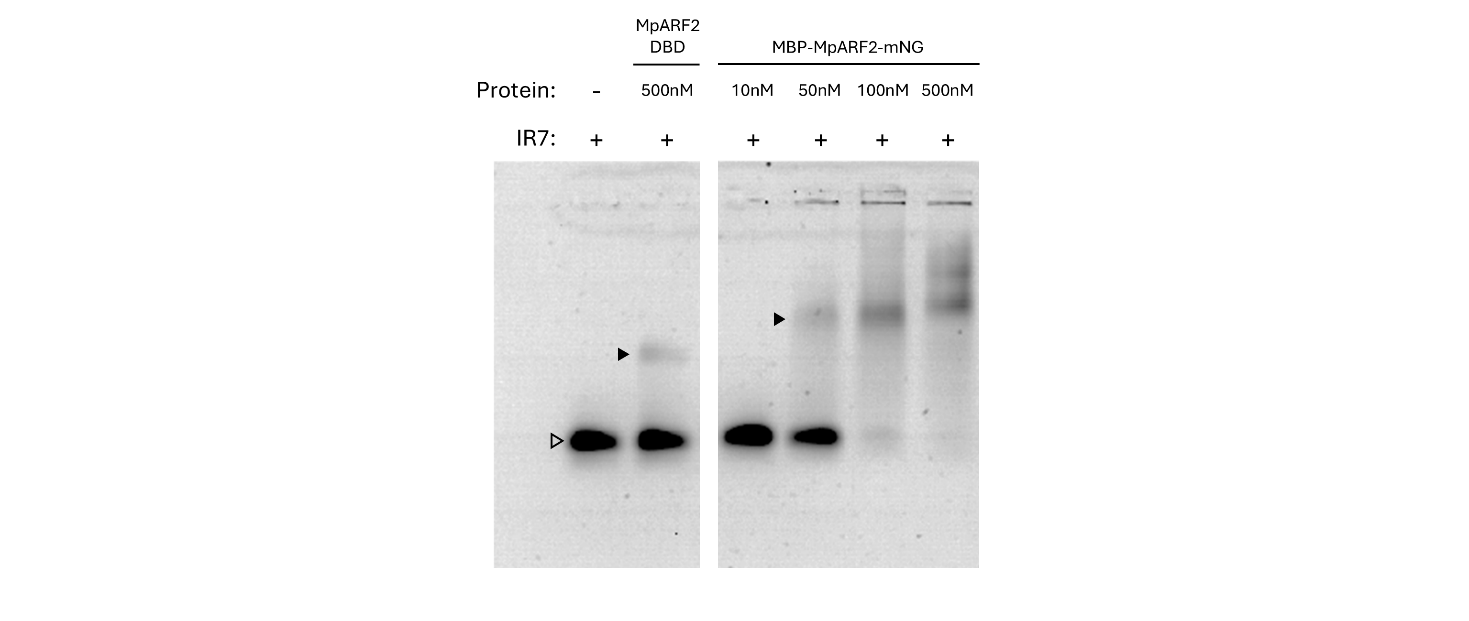
*

**Figure S3. EMSA of MpARF2-DBD and MBP-MpARF2-mNG highlighting that both proteins bind to DNA.** The open arrowhead indicates the position of unbound IR7 (37bp), the black arrowheads indicate the positions of IR7-MpARF2-DBD and IR7-MBP-MpARF2-mNG complexes, respectively.

*
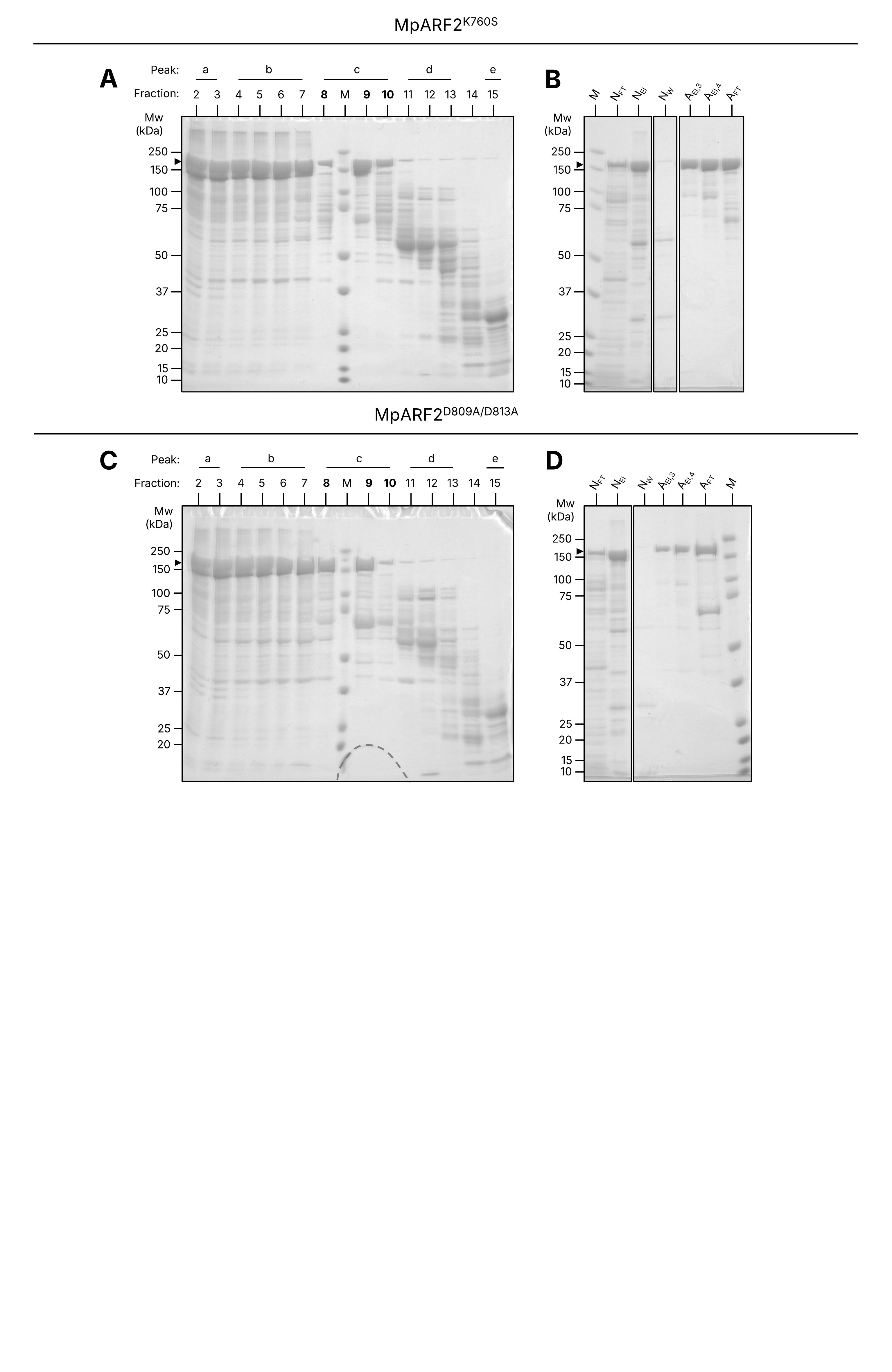
*

**Figure S4. SDS-PAGE gels of samples from the purifications of MBP-MpARF2^K760S^-mNG and MBP-MpARF2^D809A/D813A^-mNG, respectively. (A,C)** Gels corresponding to the SD200PG chromatograms of Figure **4A,B.** Fractions used for further purification are indicated in bold. **(B/D)** Gels corresponding to the Ni and amylose affinity steps of the purification. CFEs are not loaded. M: Molecular weight marker, N_FT_; Ni-column Flow-through, N_El_; Ni-column eluate, N_W_; Ni-column wash, A_El_; Amylose column eluate, A_FT_; Amylose column flow-through. Note that gel **C** is poorly polymerized around the grey dashed line, making the positions of the 15 and 10 kDa molecular weight markers unclear and preventing the lower molecular weight bands in lanes 9 and 10 from being resolved. Black arrowheads indicate ~170 kDa protein of interest.

*
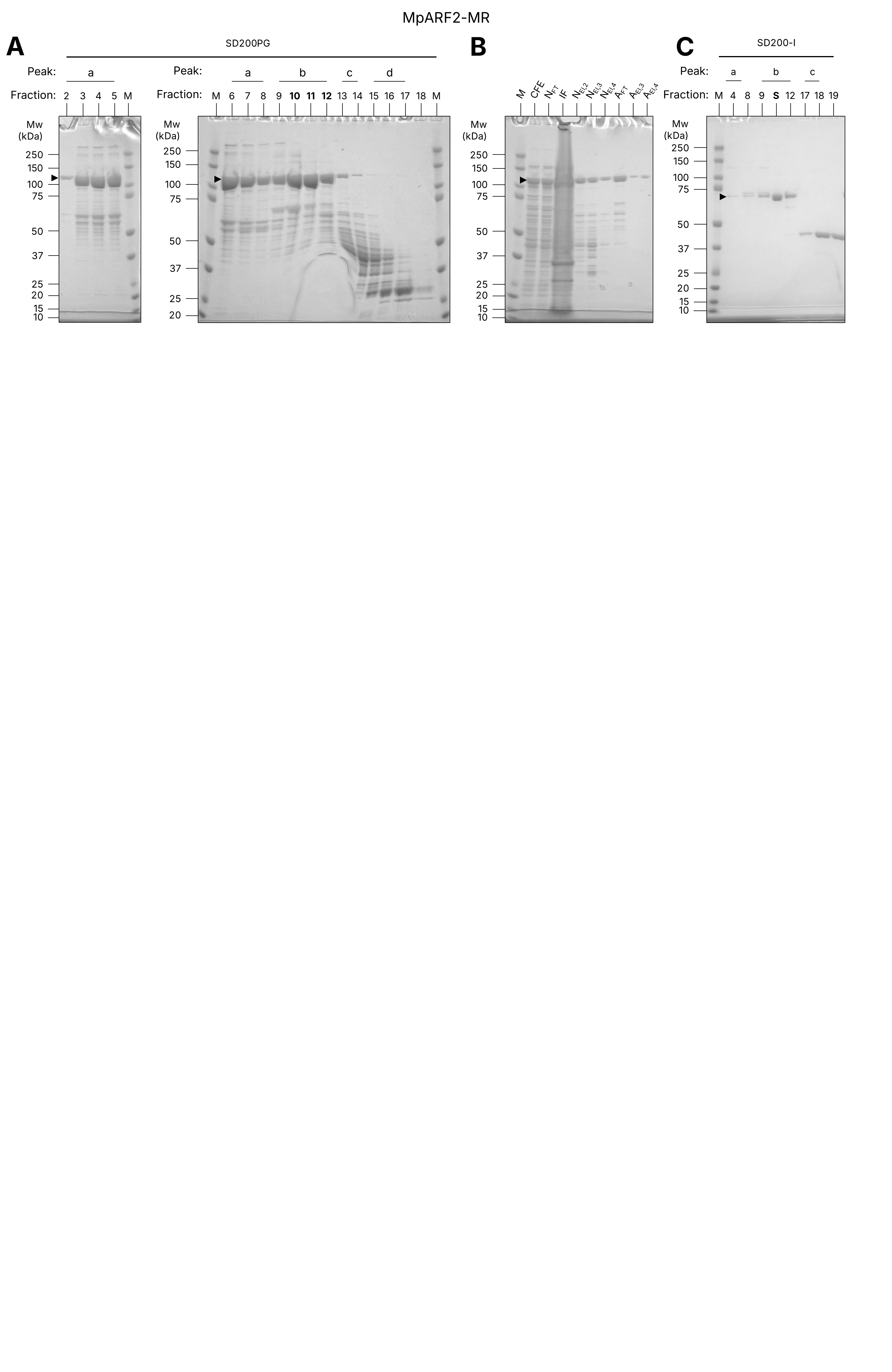
*

**Figure S5. SDS-PAGE gels of samples from the purification and preparation of (MBP-)MpARF2-MR-mNG. (A)** Gels corresponding to the SD200PG chromatogram of **Figure 5A**. Fractions used for further purification are indicated in bold. Note that the gel on the right is poorly polymerized, resulting in the lower molecular weight bands in lanes 10 through 13 being unresolved and poorly resolved in lane 14. M: Molecular weight marker. **(B)** Gel corresponding to the Ni-IMAC and amylose affinity steps of the purification. M: Molecular weight marker, CFE; Cell-Free Extract, N_FT_; Ni-column Flow-through, IF; Insoluble Fraction, N_El_; Ni-column eluate, A_FT_; Amylose column flow-through, A_El_; Amylose column eluate. **(C)** Gel corresponding to the SD200-I chromatogram of **Figure 5B.** M: Molecular weight marker, S: sample consisting of the pooled fractions 10 and 11. Black arrowheads highlight MBP-MpARF2-MR-mNG of 110 kDa **(A,B)** and MpARF2-MR-mNG of 68 kDa **(C)**.

***
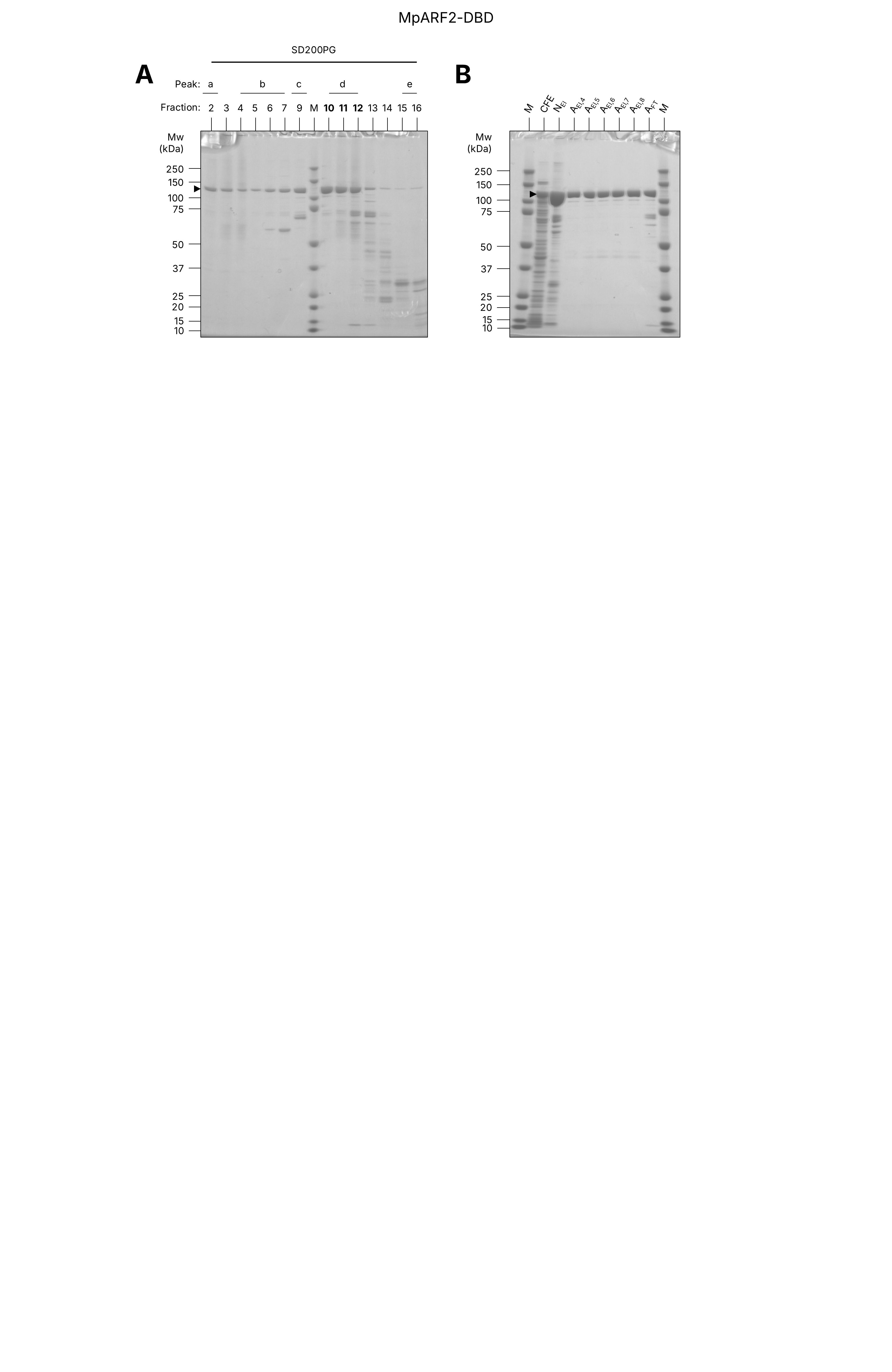
***

**Figure S6. SDS PAGE gels of samples from the purification of MBP-MpARF2-DBD-mNG. (A)** Gel corresponding to the SD200PG chromatogram of Figure 5C. Fractions used for further purification are indicated in bold. M: Molecular weight marker. **(B)** Gel corresponding to the the Ni-IMAC and amylose affinity steps of the purification. Eluates 5 to 7 are stored. M: Molecular weight marker, CFE: Cell free extract, N_El_: Ni-column eluate, A_El_: Amylose column eluate, A_FT_: Amylose column flow-through. Black arrowheads in **A,B** indicate MBP-MpARF2-DBD-mNG.

**
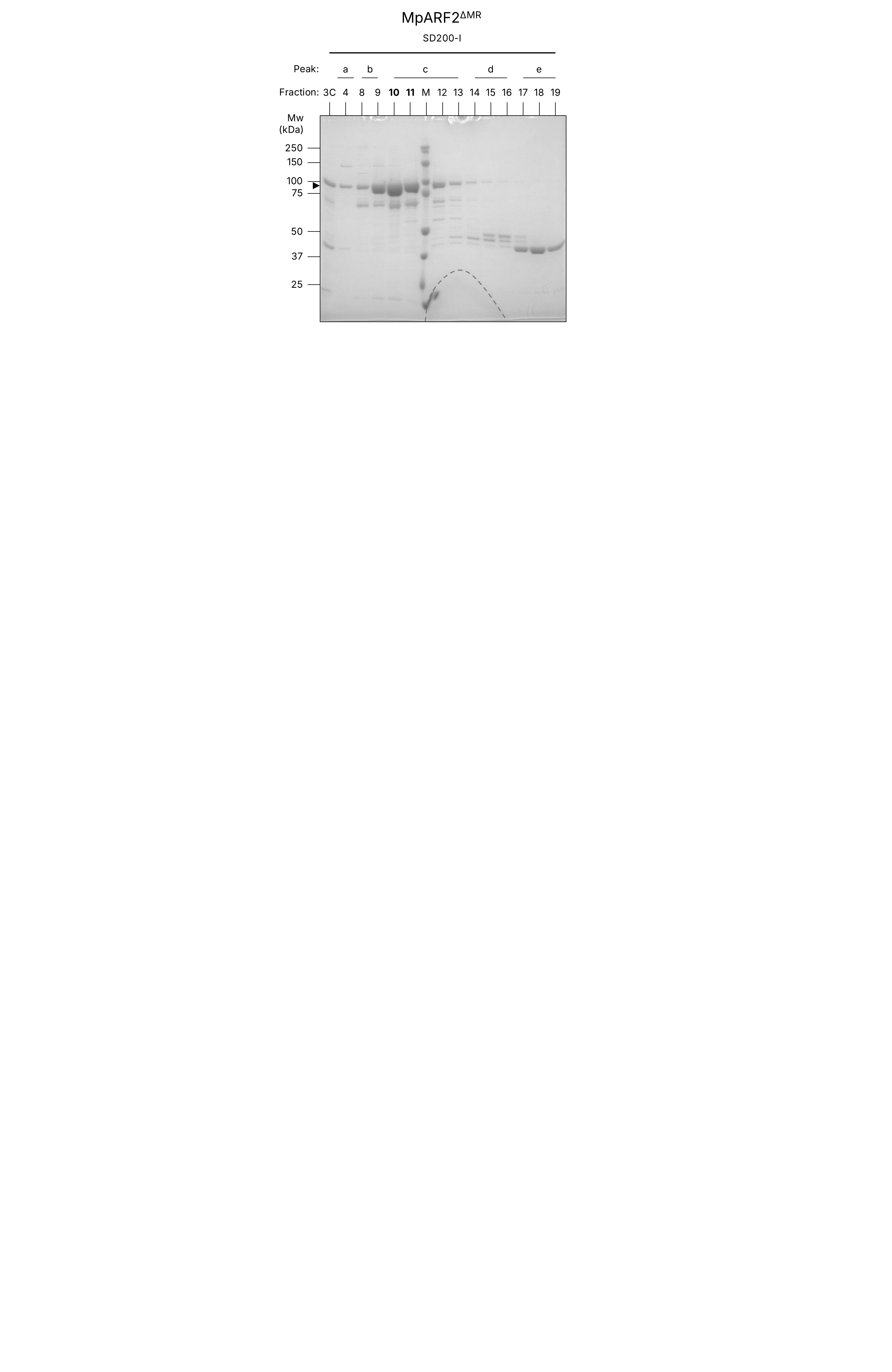
**

**Figure S7. SDS-PAGE gel of samples from the SD200-I run of MpARF2^ΔMR^-mNG.** Gel corresponding to the SD200-I chromatogram of Figure 5F. Note that the gel is poorly polymerised at the dashed line, resulting in unresolved 20, 15 and 10 kDa markers and potential lower molecular weight bands in fractions 12 through 16 being unresolved. 3C: sample after 2h incubation with 3C protease, M: Molecular weight marker. Black arrowhead indicates MBP-MpARF2^ΔMR^-mNG.

**
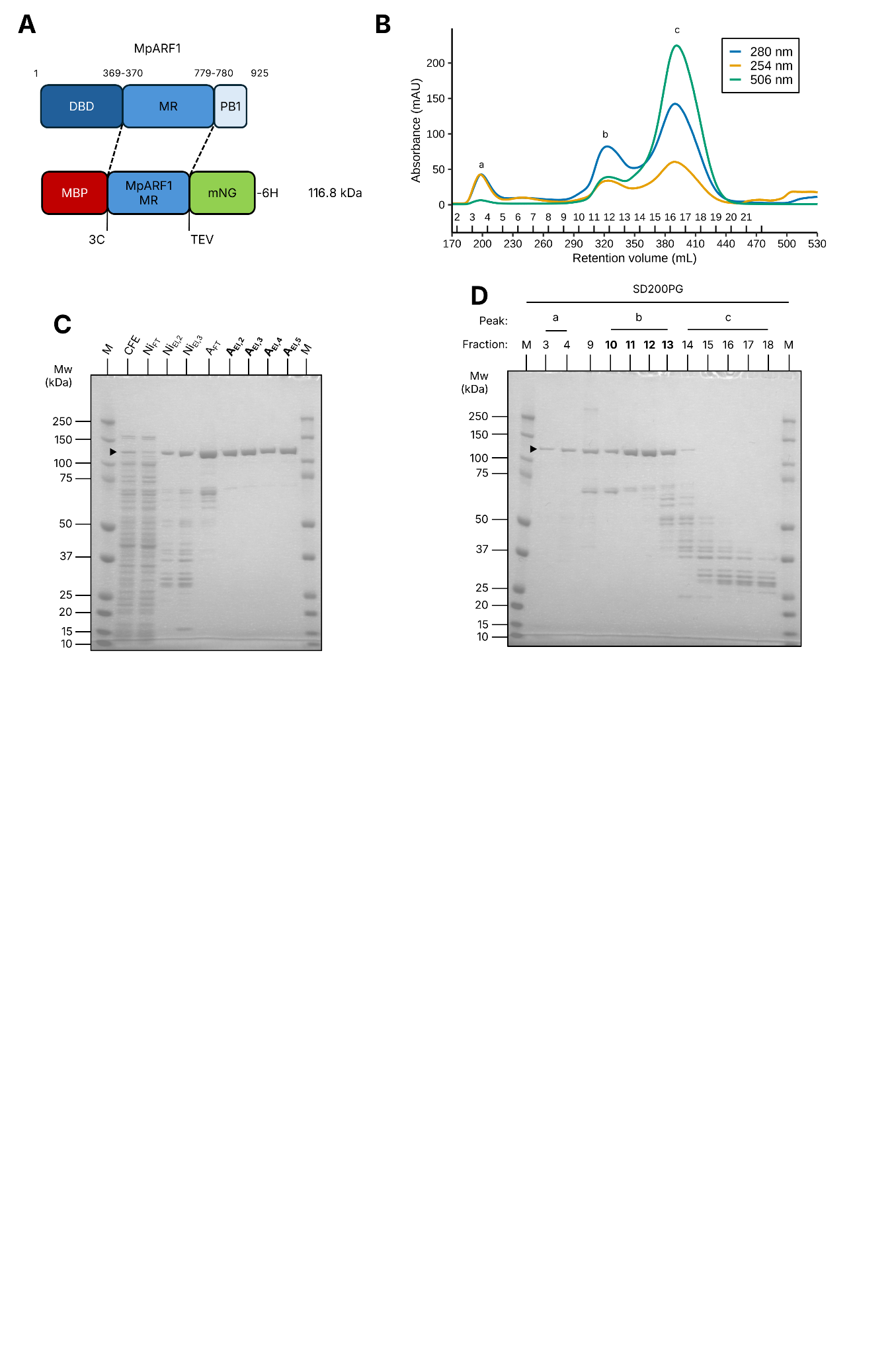
**

**Figure S8. Purification of MpARF1-MR. (A)** Graphical representation of the MpARF1 domain structure, with amino acid boundaries and the molecular weight of MBP- and mNG-tagged protein. **(B)** UV-Vis chromatograms of SD200PG SEC runs of Ni-purified MBP-MpARF1-MR-mNG. Inner tick marks indicate starting points of fractions. **(C)** SDS-PAGE gel of all non-SEC samples from the purification of MBP-MpARF1-MR-mNG. Amylose eluate fractions kept for storage are in bold. M: Molecular weight marker, Ni_FT_: Ni-column flow-through, Ni_El_: Ni-column eluate, A_FT_: Amylose column flow-through, A_El_: Amylose column eluate. **(D)** SDS-PAGE gel of fractions from the SD200PG run shown in **(B)**. Fractions used for further purification are in bold. Black arrowheads indicate the position of the 117 kDa MBP-MpARF1-MR-mNG. M: Molecular weight marker. One purification was performed, shown here.
